# Core clinical competency indicators for master of nursing specialist (anaesthesia) students in China: a delphi study

**DOI:** 10.1186/s12912-026-05018-8

**Published:** 2026-07-15

**Authors:** Xueyan Xing, Conghui Jiang, Tingting Yang, Jiamin Kang, Jiale Hu, Zhifeng Gao, Shumin Tu

**Affiliations:** 1 Department of Anesthesiology, Beijing Tsinghua Changgung Hospital, School of Clinical Medicine,Tsinghua Medicine, Tsinghua University, Beijing, 102218 China; 2 Department of Anesthesiology and Perioperative Medicine, Zhengzhou Central Hospital Affiliated to Zhengzhou University, Zhengzhou, 450007 China; 3 Department of Anesthesiology Department, Sichuan Provincial People’s Hospital, School of Medicine, University of Electronic Science and Technology of China, Chengdu, 610072 China; 4 School of Nursing, Xuzhou Medical University, Xuzhou, 221004 China; 5 Virginia Commonwealth University, Richmond, VA 23298 USA

**Keywords:** Anaesthesia nursing, Master, Professional degree, Clinical practice, Competence, Competency, Education

## Abstract

**Background:**

Competency training is essential for cultivating qualified anaesthesia Master of Nursing Specialist (MNS) graduates. Although a previous study has established relevant competency dimensions for anaesthesia nursing, no refined framework with operationalized indicators is currently available for Chinese MNS anaesthesia students.

**Objective:**

To construct an index system for the core clinical competencies of MNS anaesthesia students.

**Methods:**

Following literature screening, 26 eligible publications were selected to draft preliminary indicators. Semi-structured interviews with 15 participants were performed, and interview data were analysed via thematic coding.21 experts from 11 regions across China completed two rounds of Delphi surveys. Indicators were rated on a 5-point Likert scale, with expert consensus determined by predefined statistical cut-offs (mean importance score ≥ 3.5, coefficient of variation (CV) ≤ 0.25, ≥ 75% ratings ≥ 4) combined with comprehensive expert revision opinions.

**Results:**

The response rates of the two Delphi rounds were 100% and 95.24%, respectively. The expert authority coefficients (Cr) were 0.95 and 0.96, indicating high reliability of the consultation results. In the second round, Kendall’s concordance coefficients (W) for the first, second, and third-level indicators were 0.552 (strong consensus), 0.324 (moderate consensus), and 0.254 (weak-moderate consensus) respectively (*P* < 0.01), which indicated that the consistency of expert opinions was statistically significant and the consultation results were valid. The range of coefficient of variation was from 0.05 to 0.25. The final indicator system included 8 first-level indicators, 39 s-level indicators, and 109 third-level indicators.

**Conclusion:**

Core clinical competency indicators for MNS anaesthesia students, established in this study, achieved expert consensus validity. These competency indicators can serve as a theoretical reference for the future training and assessment of MNS postgraduates in this field.

**Supplementary Information:**

The online version contains supplementary material available at https://doi.org/10.1186/s12912-026-05018-8.

The rapid advancement of surgical and anaesthetic techniques has placed increasing demands on the clinical competence of anaesthesia nursing staff. Such competence is widely recognised as a cornerstone of perioperative patient safety [1]. Nursing education, in turn, plays a critical role in developing these competencies and thereby advancing patient safety outcomes [2]. Anaesthesia nursing education originated in the United States, transitioning from hospital-based training to university-based graduate programmes in 1998. Evidence suggests that advanced practice nurses (APNs) trained through such programmes contribute positively to patient safety outcomes [3]. In China, the MNS programme in anaesthesia serves as a key avenue for developing APNs in anaesthesia [4].

Clinical practice is a crucial component of nursing education. During clinical practice, students integrate and apply professional knowledge while developing clinical technical and non-technical skills [5, 6].Although anaesthesia nursing education in China started relatively late, some universities have already enrolled MNS students in anaesthesia [7]. In addition to mastering the professional knowledge, MNS students in anaesthesia in China must complete clinical practicum training, and the required duration of clinical practice must account for no less than 50% of the total duration of the MNS programme. Nevertheless, most MNS anaesthesia students still fall short of employers’ expectations in clinical competence after graduation [8].

At present, no refined core clinical competency indicator system adapted for MNS anaesthesia students in China, which is essential for competency-based education (CBE) and the cultivation of anaesthesia nursing specialists. The International Federation of Nurse Anaesthetists (IFNA) has only put forward 27 international clinical practice objectives without localized adaptive contents [9]. Hu et al. [8] identified six competency dimensions for MNS anaesthesia students through qualitative inquiry, providing an important foundation. However, their study did not operationalise these dimensions into measurable indicators with specific behavioural descriptors – a necessary step for objective assessment. While existing studies provide valuable reference points [10, 11, 12], they require substantial localization to China’s social, cultural, and regulatory context. Moreover, none have produced a comprehensive, multi-level indicator system with detailed behavioural descriptors suitable for direct clinical assessment. A China-specific core clinical competency indicator system for MNS anaesthesia students, therefore, remains to be developed.

The lack of core clinical competency indicators as guidance and training objectives in the cultivation of MNS anaesthesia students will lead to unbalanced ability development, difficulty in meeting graduation requirements, and even an increased risk of adverse patient safety events [13, 14].Therefore, it is urgent to adopt scientific methods to establish a comprehensive core clinical competency indicator system, so as to facilitate the development of talent training for MNS anaesthesia students.This study aims to construct a core competency indicator system for clinical practice of MNS anaesthesia students, so as to provide references for the development of anaesthesia nursing master’s programme.

## Theoretical framework

Developed by the International Federation of Nurse Anaesthetists (IFNA) in 2016 based on the Canadian Medical Education Directives for Specialists (CanMEDS) framework [15], the Nurse Anaesthetist Role Model was formally adopted in the International Council of Nurses (ICN) *Guidelines on Advanced Practice Nursing – Nurse Anaesthetists* in 2021 [16]. This model defines seven core professional roles for nurse anaesthetists, among which the Nurse Anaesthesia Expert serves as the central integrative role that incorporates the requirements of the other six complementary roles into standardised clinical practice standards, rather than functioning as an independent substitute [15]. Given that the core training objective of Chinese anaesthesia MNS programmes is to cultivate specialised clinical nurse anaesthesia experts [8, 13], this study constructed a clinical competency indicator system centred on the Nurse Anaesthesia Expert role. The remaining six CanMEDS roles, although essential for professional development, primarily represent generic competencies including communication, collaboration, and leadership, which are already covered in domestic undergraduate nursing education [12]. Therefore, core components of these six roles are embedded into the system as supportive competencies, ensuring the entire indicator system focuses on the specialised clinical capabilities required for anaesthesia MNS students and prevents scattered assessment priorities.

## Methods

### Design

The Delphi method is a group consensus technique. Its overall purpose is to synthesize expert opinions into a consensus. The main characteristics of the Delphi method are participant anonymity, iteration, controlled feedback, and statistical group response [17, 18].This study adopted the Delphi process to solicit experts’ opinions on the indicators for the core clinical practice competencies of MNS anaesthesia students. The study consisted of two phases: (1) Development of the preliminary draft of indicators: The preliminary draft of the indicators of the core clinical practice competencies of MNS anaesthesia students was developed through literature review and semi-structured interviews. (2) Delphi phase: Based on the preliminary draft, a Delphi questionnaire was designed, and two rounds of Delphi surveys were conducted to reach a consensus.

### Formation of the research team

The research team comprised eight members: two clinical anaesthesia experts, two clinical anaesthesia nursing experts, two anaesthesia nursing education experts and two MNS anaesthesia students, including five senior and one intermediate title holders. The principal investigator is the nursing director of the anaesthesiology department and a master’s supervisor in anaesthesia nursing at a grade A tertiary hospital, with 35 years of clinical experience and expertise in nursing research methodology. The team was responsible for preliminary research, Delphi questionnaire design, distribution and collection, expert recruitment, collation and analysis of expert opinions, as well as revision of the indicator system according to expert feedback. The two MNS anaesthesia students did not participate in expert selection or the final decision-making of indicator screening throughout the entire study to avoid potential conflicts of interest.

### Development of the first draft of the clinical practice competency indicators for MNS anaesthesia students

We searched PubMed, Web of Science, Embase, CINAHL, Chinese Medical Association Journal Full-text Database, China National Knowledge Infrastructure (CNKI), China Science and Technology Journal Database (VIP) and Wanfang Database from the inception of each database to December 2024, using the search terms “nurse anaesthetists”, “nurse anaesthesia”, “anaesthesia nurse”, “anaesthesia nursing”, “certified registered nurse anaesthetist”, “professional competence”, “core competency”, “core competencies”, “competence” and “competency”. Articles without available full texts, publications in languages other than Chinese or English, and studies irrelevant to the research topic were excluded. A total of 793 records were initially retrieved, among which 178 duplicate articles were removed. The remaining records were screened by titles and abstracts according to the predefined inclusion and exclusion criteria, 94 articles were further assessed for full-text eligibility, of which 68 were excluded (reasons provided in Additional file 1), and ultimately 26 eligible studies were included. Full search strings and the literature screening flowchart are presented in Additional file 1. In addition, this study also referred to the educational and practice standards for nurse anaesthesia established by the Council on Accreditation of Nurse Anesthesia Educational programmes [19] and the International Federation of Nurse Anaesthetists (IFNA) [16], the core requirements for master’s education in nursing issued by the American Association of Colleges of Nursing (AACN) [20] and IFNA [9], as well as the professional degree postgraduate education development plan and training objectives for Master of Nursing Specialist formulated by the Ministry of Education of China [21, 22].

The final interview outline was formulated based on comprehensive literature review and expert group discussions, with the complete interview questions presented in Additional file 2. Participants were enrolled via purposive sampling and categorized into three groups: third-year students enrolled in the anaesthesia track of the Master of Nursing Specialist (MNS) programme, MNS anaesthesia programme supervisors, and clinical preceptors. The inclusion criteria were strictly defined as follows: student participants must be registered third-year MNS students specializing in anaesthesia; programme supervisors were required to hold a master’s degree or above, possess an associate senior or higher professional title, and have accumulated no less than 10 years of nursing education experience; clinical preceptors must hold a bachelor’s degree or higher, obtain an intermediate or above professional title, and have more than 10 years of clinical working experience in anaesthesia nursing. From January to February 2025, one systematically trained researcher (the fourth author) conducted one-on-one semi-structured interviews in a quiet and private environment, with each interview lasting 30 to 45 min. Prior to data collection, the researcher fully elaborated the study objectives, interview contents, research procedures, interview duration, as well as potential benefits and risks to all participants. Formal interviews were initiated only after participants had fully understood and agreed to the relevant research arrangements. A total of 15 participants were finally recruited, including five third-year anaesthesia MNS students, five programme supervisors, and five clinical preceptors. Two researchers independently reviewed interview transcripts after each set of three interviews; saturation was defined as three consecutive interviews yielding no new codes, consistent with established qualitative research guidelines [23]. Participant recruitment was terminated when data saturation was achieved, which was confirmed by the absence of new themes in three consecutive interviews following the 15th interview.

Through semi-structured interviews, we extracted all themes identified in the literature review stage and identified a new theme: clinical nursing research. Accordingly, a preliminary draft of core competency indicators for clinical practice among anaesthesia MNS postgraduates was established, including 10 first-level indicators, 33 s-level indicators, and 104 third-level indicators.

### Delphi process

#### Expert panel selection

Inclusion criteria for expert consultants were as follows: (1) Master’s degree or above, associate senior professional title or higher, with more than 10 years of experience in nursing education; (2) Bachelor’s degree or above, intermediate professional title or higher, with more than 10 years of experience in clinical anaesthesia nursing or anaesthesia nursing management; (3) Master’s degree or above, associate senior professional title or higher, with more than 10 years of experience in clinical anaesthesia practice. Experts who met any of the above criteria and voluntarily participated in this study were included. Considering the disciplinary representativeness and geographical distribution of experts, a purposive sampling method was used to recruit experts from different regions in China.

#### Data collection

In Round 1, the research team developed a questionnaire based on the initial draft, consisting of three parts(see Additional file 3). The first part was a letter to the experts, which mainly explained the background, purpose, and significance of the study. The second part was the consultation form and completion instructions for the core competency indicators of clinical practice for MNS anaesthesia students. It included 10 first-level indicators, 33 s-level indicators, and 104 third-level indicators. The importance of each indicator was assessed by a 5-point Likert scale, ranging from 5 (“very important”) to 1 (“totally unimportant”) rating scale. Experts rated each indicator and could provide comments on modification, addition, or deletion, as well as propose additional indicators not included in the initial version. The third part was the expert information form, which included the general information, professional title, research field, working years, familiarity coefficient (Cs) and judgment coefficient (Ca). Cs reflected the experts’ familiarity with the survey content and was divided into five levels: 0.20 = unfamiliar, 0.40 = less familiar, 0.60 = generally familiar, 0.80 = very familiar, 1.00 = extremely familiar. Ca represented the experts’ judgment basis, covering four categories: theoretical analysis, practical experience, domestic and international literature, and intuitive judgment. The consulted experts were unaware of each other and communicated only with the research team.

After the first-round questionnaires were collected, the research team added, deleted, or revised indicators according to the indicator screening criteria and expert opinions. The criteria for indicator screening were as follows: mean importance score ≥ 3.5, coefficient of variation (CV) ≤ 0.25. Indicators with mean < 3.5 or CV > 0.25 were subjected to collective review by the research team. Only those indicators recognized by more than 75% of the team members as having clinical practical value were retained [24]. In Round 2, Experts completed the revised questionnaire in the same way(see Additional file 4). Questionnaires in each round were distributed via email or WeChat. Each expert received a unique code. A separate, password-protected file linking codes to names was accessible only to the principal investigator for the sole purpose of sending reminders. All other team members accessed only de-identified data. All questionnaires were collected within two weeks. A reminder was sent via email or WeChat if the questionnaire was not returned within 10 days.The first round was conducted from March 10 to March 24, 2025, and the second round was conducted from April 7 to April 21, 2025.

#### Data analysis

Conventional content analysis was applied for the data analysis of semi-structured interviews. The coding procedure was independently conducted by two trained researchers to guarantee the rigor of data analysis. Data saturation was assessed throughout the interview and analytical process. Participant recruitment was terminated when no new themes emerged in three consecutive interviews, confirming that the collected data were sufficiently comprehensive and adequate for thematic analysis.

Microsoft Excel 2013 and SPSS 22.0 were used for statistical analysis of quantitative data. The general characteristics of experts were described using frequency, percentage, and mean ± standard deviation (SD). Although Likert data are ordinal, means and SDs are commonly reported in Delphi studies to facilitate comparison [25].The expert positive coefficient was reflected by the questionnaire response rate, and a response rate > 70% was regarded as satisfactory [24]. The authority of experts was measured by the authority coefficient (Cr), which was calculated based on the judgment coefficient (Ca) and familiarity coefficient (Cs), using the formula: Cr = (Ca + Cs) / 2. Results were generally considered reliable when Cr ≥ 0.70 [26]. The coordination degree of expert opinions was expressed by Kendall’s concordance coefficient (Kendall’s W) and coefficient of variation (CV). The significance level for Kendall’s W was set at 0.05 [17]. A higher Kendall’s W value indicated better coordination of indicators and higher consistency among experts. Indicators were identified as possessing significant research value if ≥ 75% of all experts assigned a score of 4 or higher [25]. Content Validity Ratio (CVR) and Content Validity Index (CVI) were not calculated in this study, as expert consensus obtained via the Delphi method was adequate to reflect content validity.

### Ethical considerations

This study was performed in strict accordance with the ethical principles outlined in the Declaration of Helsinki and approved by the Ethics Committee of Beijing Tsinghua Changgung Hospital (Approval No. 24794-4-01). Written informed consent was obtained from all participants prior to data collection. All participants were fully informed of their right to withdraw from the research at any time without penalty. All identifying information was stripped from interview transcripts and Delphi questionnaires, and all data were accessible only to members of the research team.

## Results

### Characteristics of the experts

A total of 21 experts were included, from 5 universities and 12 hospitals (all university-affiliated hospitals) across 10 different regions in China. Among them, 6 were male and 15 were female, the age ranged from 37 to 59 years (mean = 48.05, SD = 6.94), and the working experience ranged from 10 to 40 years (mean = 23.75, SD = 9.06). Nine held doctoral degrees, 1 held a master’s degree, and 11 held bachelor’s degrees. Nine had senior professional titles, and 12 had associate senior professional titles. Additional demographic and professional characteristics are presented in Table 1. Finally, 21 experts completed the first round of consultation, and 20 experts completed the second round.

**Table 1:** Characteristics of the experts

Characteristic	*N*(%)
Gender	
Male	6(28.6%)
Female	15(71.4%)
Age	
30–39	3(14.3%)
40–49	9(42.9%)
50–59	9(42.9%)
Working experience	
10–19	7(33.3%)
20–29	7(33.3%)
30–40	7(33.3%)
Degree	
Doctor	9(42.8%)
Master	1(4.8%)
Bachelor	11(52.4%)
Professional title	
Senior	9(42.9%)
Associate Senior	12(57.1%)
Current position	
Clinical Nursing Educator	3(14.3%)
Head Nurse	4(19.0%)
Anaesthesiologist	1(4.8%)
anaesthesia master’s degree supervisors	6(28.6%)
anaesthesia nursing master’s degree supervisors	7(33.3%)
Region	
Beijing	6(28.6%)
Guangdong	3(14.3%)
Jiangsu	1(4.8%)
Henan	2(9.5%)
Hunan	1(4.8%)
Anhui	1(4.8%)
Sichuan	1(4.8%)
Hubei	1(4.8%)
Fujian	3(14.3%)
Jiangxi	1(4.8%)
Taiwan, Province of China	1(4.8%)

### Expert enthusiasm and authority

The effective response rate of the first round of consultation was 100% (21/21), and 12 experts (57.14%) provided revised opinions. The effective response rate of the second round was 95.24% (20/21), and 10 of the 20 responding experts (50%) provided revised opinions. The scores for experts’ degree of familiarity and judgment basis for the indicators are shown in Additional file 5. The authority coefficients of experts in the two rounds are presented in Table 2.

**Table 2:** The authority coefficients of experts

Item	Round1		Round2	
	Mean	Standard Deviation	Mean	Standard Deviation
Familiarity coefficient	0.94	0.13	0.96	0.10
Judgment coefficient	0.95	0.07	0.96	0.06
Authority coefficient	0.95	0.07	0.96	0.06

### Degree of coordination of expert opinions

In the first round, Kendall’s concordance coefficients (Kendall’s W) for the first-level, second-level, and third-level indicators were 0.302, 0.358, and 0.191, respectively. In the second round, the values were 0.552, 0.324, and 0.254. All coefficients were statistically significant (*P* < 0.01), as detailed in Table 3. Kendall’s W values between 0.2 and 0.4 indicate moderate consensus; values > 0.5 indicate strong consensus (round 2 first-level indicators), consistent with conventional Delphi consensus grading standards [25]. In the second round, the mean importance scores for the core competency indicators of MNS anaesthesia students in clinical practice ranged from 4.05 to 4.95, with coefficients of variation ranging from 0.05 to 0.25(see Additional file 6). This indicates a high level of consensus among experts regarding the importance of these core competency indicators for MNS anaesthesia students.

**Table 3:** Kendall’s concordance coefficients for the two rounds of consultation

Stage	Indicator	Kendall’s W	x^2^	*P*
Round 1	Primary Indicator	0.302	57.005	0.000
	Secondary Indicator	0.358	240.407	0.000
	Tertiary Indicator	0.191	413.225	0.000
Round 2	Primary Indicator	0.552	77.276	0.000
	Secondary Indicator	0.324	245.926	0.000
	Tertiary Indicator	0.254	549.505	0.000

### Results of Delphi expert consultation

#### Round 1 consultation

In the first round of expert consultation, indicator structure was optimised via merging, revision, addition and deletion. The initial framework contained 10 primary, 33 secondary, and 104 tertiary indicators; after revision, the system consisted of 8 primary, 39 secondary, and 109 tertiary indicators. Three primary indicators were consolidated into a single dimension named Clinical anaesthesia nursing, and wording refinements were applied to four other primary indicators. For secondary indicators, one item was deleted, eight new items (including body temperature monitoring and neuromuscular transmission function monitoring) were supplemented, two overlapping items were integrated, and seven items received textual revisions. One tertiary indicator was removed, six tertiary items were newly added, and six tertiary indicators were revised in phrasing. A full item-by-item comparison between the pre- and post-consultation indicator frameworks is presented in Additional File 7.

#### Round 2 consultation

In the second round of consultation, no adjustments were made to the primary or secondary indicators. Only one tertiary indicator was revised: “Know the principles of perioperative pain management” was updated to “Know the principles of perioperative acute pain management”.

The finally established Core clinical competency indicators for anaesthesia MNS postgraduates consist of 8 primary indicators, 39 secondary indicators and 109 tertiary indicators. Detailed items are presented in Additional File 6.

## Discussion

Anaesthesia nursing ranks as one of the earliest established specialties for international advanced practice nurses (APNs). In most nations and territories, anaesthesia APNs must hold a master’s degree for entry to practice [15]; the American Association of Nurse Anaesthetists (AANA) stands apart, having set a doctoral degree as the entry criterion for Certified Registered Nurse Anaesthetists (CRNAs) from 2025 onwards. Clinical competency is widely acknowledged as a fundamental prerequisite for safe perioperative care [27], and our proposed framework can boost patient safety indirectly through directing competency-based education. Critically, a standardised core competency indicator system for anaesthesia MNS students is absent both in China and globally, which creates an urgent imperative to construct such a system.

This study built a localized multi-level competency indicator system for anaesthesia nursing postgraduates. We adopted only the Expert role from the CanMEDS framework as the core theory. Combined with IFNA’s competency standards for anaesthesia nurse specialists, literature review, interviews and Delphi consultation, we redefined relevant competencies, carried out localized adjustments and hierarchical indicator design. This work adapts and extends international theories for domestic anaesthesia nursing education with unique theoretical application value.

A total of 21 experts were recruited from 5 universities and 12 grade A tertiary hospitals, all with senior professional titles. Their backgrounds covered nursing education, anaesthesia nursing management, and clinical anaesthesiology, and 15 (71.43%) had over 20 years of work experience, ensuring rich experience and good representativeness. The expert authority coefficients were 0.95 and 0.96 (both > 0.70) in the two rounds, indicating high professional authority. The effective questionnaire response rates reached 100% and 95.24%, with opinion proposal rates of 57.14% and 50%, reflecting strong expert enthusiasm [28]. Kendall’s concordance coefficients verified good consensus among experts. Furthermore, strong consensus was defined as ≥ 75% of experts scoring an indicator ≥ 4 points, which further strengthened the reliability of the results.

The indicator system constructed in this study covers the entire peri-anaesthesia nursing process, including preoperative, intraoperative, and postoperative care. It fully contains core competencies involving clinical operation, safety management, infection prevention and control, documentation, risk management and clinical research. This system conforms to clinical practical demands and matches the training objectives of MNS anaesthesia students, who are required to have solid clinical practical capabilities and scientific research literacy [8]. Unlike Yin’s undergraduate competency system, which focuses on foundational skills, our system incorporates advanced competencies such as clinical research and risk management, reflecting the higher-order expectations of master’s-level graduates. Our Delphi results further operationalized Hu’s general competencies into measurable sub-indicators, such as standardized airway management skills. In addition, Unlike Sanclemente-Dalmau’s general nurse anaesthetist competencies, our system is tailored to MNS anaesthesia students and includes detailed third-level behavioral indicators. Furthermore, we incorporated a ‘clinical nursing research’ dimension, which is often missing from clinical competency frameworks.

Pre-anaesthesia nursing assessment ranks foremost among prioritised competencies owing to its capacity to mitigate anaesthetic hazards and secure patient safety [10]. Formulating individualised anaesthesia care plans via the nursing process after pre-anaesthetic assessment shifts care forward to the preoperative period and embodies holistic nursing principles. The clinical anaesthesia nursing domain covers full perioperative workflows: intraprocedural expertise, secure postoperative handover and transport, plus PACU surveillance and care. Reliable postoperative transfer and handoff act as critical safeguards for perioperative safety [29], while PACU monitoring and nursing constitute an irreplaceable segment of anaesthesia nursing practice [30].

Infection prevention and control underpins all safe anaesthesia nursing delivery [31], and robust risk management elevates team emergency proficiency and institutional safety culture [32]. As future disciplinary leaders, anaesthesia nursing MNS trainees will drive ward safety system development, making targeted risk management training a high priority in postgraduate curricula [33].

Anaesthesia monitoring received high importance ratings (mean ≥ 4.65 for all circulatory and respiratory indicators). Anaesthesia monitoring provides important supporting information for clinical decision-making and is also an essential part of anaesthesia nursing practice [7, 34]. The application of instruments and equipment, as well as anaesthesia documentation, are also indispensable components of anaesthesia nursing [35, 36]. Distinction should be made between competency requirements for basic equipment and advanced specialized equipment. In addition, the relatively low consensus on neuromuscular monitoring (CV up to 0.25) may reflect regional variability in equipment availability or practice scope. Future revisions should consider splitting this indicator into basic vs. advanced levels.

The dimension of clinical nursing research reflects the integration of research competence and clinical ability among MNS anaesthesia students, and is also one of the core competencies of APNs [37]. It helps promote the transformation of MNS anaesthesia students into anaesthesia APNs [38].

Despite these strengths, several limitations should be acknowledged.

### Limitations

This study presents several limitations. Geographically, most Delphi experts were from southeastern China, causing potential regional bias, and the developed competency indicator system remains insufficiently verified empirically. It is necessary to popularize the system in clinical teaching and student assessment to enhance its practicability. In addition, voluntary expert participation may trigger self-selection bias and overestimate research consensus. Experts may have been influenced by social desirability, particularly for indicators derived from international guidelines, potentially inflating importance ratings. This study also failed to involve employers and patients, hence multi-stakeholder perspectives should be included in future studies. Given that all research tools were designed in Chinese, the findings are mainly applicable to domestic settings, and cross-cultural adaptation is required for international promotion.

## Conclusions

This study established a Core clinical competency indicator system for MNS anaesthesia students using the Delphi method.The final system comprises 8 primary indicators, 39 secondary indicators, and 109 tertiary indicators covering the full peri-anaesthesia nursing process.

This indicator system can serve as a structured competency framework for the training of MNS anaesthesia students. It can help universities formulate training plans and optimize curricula, enable clinical preceptors to evaluate students’ practical abilities, and provide a basis for relevant institutions to establish unified professional competency standards. For universities, the hierarchical structure can guide course sequencing; for clinical preceptors, the tertiary indicators can serve as observational checklists during clinical rotations; for professional bodies, the system offers a foundation for national certification standards. Further studies are needed to verify its actual educational effects. Pilot testing in multiple anaesthesia MNS training programmes is recommended to confirm feasibility and validity. Moreover, future research should conduct prospective validation linking the competency indicators to clinical performance metrics and patient safety outcomes. The research team will develop a user guide and assessment rubric to facilitate adoption by clinical educators, and will evaluate the system’s usability in a subsequent implementation study.

## Supplementary Information

Below is the link to the electronic supplementary material.

Supplementary Material 1

Supplementary Material 2

Supplementary Material 3

Supplementary Material 4

Supplementary Material 5

Supplementary Material 6

Supplementary Material 7

## Data Availability

The data that support the findings of this study are available from the corresponding author upon reasonable request.

## Declarations

This Delphi study did not undergo prospective trial registration.

## Abbreviations

MNS: Master of Nursing Specialist
APNs: Advanced Practice Nurses
IFNA: International Federation of Nurse Anaesthetists
CBE: Competency-based Education
CanMEDS: Canadian Medical Education Direction System
ICN: International Council of Nurses
CNKI: China National Knowledge Infrastructure
AACN: American Association of Colleges of Nursing
CV: Coefficient of Variation
Cr: Authority coefficient
Ca: Judgment coefficient
Cs: Familiarity coefficient
SD: Standard Deviation
PACU: Post-anaesthesia Care Unit
ICU: Intensive Care Unit
ECG: Electrocardiography
ASA: American Society of Anaesthesiologists
NBP: Non-invasive Blood Pressure
ABP: Arterial Blood Pressure
CVP: Central Venous Pressure
BIS: Bispectral Index
rSO₂: regional cerebral oxygen saturation
CRNAs: Certified Registered Nurse Anaesthetists
