# Supplementary material for: Core clinical competency indicators for master of nursing specialist (anaesthesia) students in China: a delphi study
Source: BMC Nurs. 2026 Jul 15;25:947. doi: 10.1186/s12912-026-05018-8 (PMC13651016; doi:10.1186/s12912-026-05018-8)
Supplement: Supplementary file 1 [file 12912_2026_5018_MOESM1_ESM.docx]

1. **Full search strings with Boolean operators**

("nurse anesthetists" OR "nurse anesthesia" OR "anesthesia nurse" OR "anesthesia nursing" OR "certified registered nurse anesthetist" OR "CRNA") AND ("professional competence" OR "core competency" OR "core competencies" OR "competence" OR "competency")

2.**Database searching**

(关键词=麻醉护士 OR 麻醉护理 OR 麻醉科护士 OR 麻醉专科护士*) AND 关键词=核心能力 OR 胜任力*

(题名或关键词:(麻醉护士 or 麻醉护理 or 麻醉科护士 or 麻醉专科护士) and 题名或关键词:(核心能力 or 胜任力))

KY=('麻醉护理' + '麻醉护士' + '麻醉科护士' + '麻醉专科护士') AND KY=('核心能力' + '胜任力')

K=(麻醉护理 OR 麻醉护士 OR 麻醉科护士 OR 麻醉专科护士) AND K=(核心能力 OR 胜任力)

Search: ("nurse anesthetists" OR "nurse anesthesia" OR "anesthesia nurse" OR "anesthesia nursing" OR "certified registered nurse anesthetist" OR "CRNA") AND ("professional competence" OR "core competency" OR "core competencies" OR "competence" OR "competency") Filters: from 1975 - 2024

TS=("nurse anesthetists" OR "nurse anesthesia" OR "anesthesia nurse" OR "anesthesia nursing" OR "certified registered nurse anesthetist" OR "CRNA") AND TS=("professional competence" OR "core competency" OR "core competencies" OR "competence" OR "competency")

("nurse anesthetists" OR "nurse anesthesia" OR "anesthesia nurse" OR "anesthesia nursing" OR "certified registered nurse anesthetist" OR "CRNA") AND ("professional competence" OR "core competency" OR "core competencies" OR "competence" OR "competency")

("nurse anesthetists" OR "nurse anesthesia" OR "anesthesia nurse" OR "anesthesia nursing" OR "certified registered nurse anesthetist" OR "CRNA") AND ("professional competence" OR "core competency" OR "core competencies" OR "competence" OR "competency")

**3.Literature screening fowchart**

Records obtained from the preliminary search

(n=793)

Records screened

(n=615)

Full-text article assessed for eligibility

(n=94)

Final inclusion of literature

(n=26)

Records after duplicates and were excluded

(n=178)

Records excluded based on title and abstarct

(n=521)

Full-text unavailable(n=14)

Languages other than chinese and english(n=3)

Irrelevent to the research question(n=51)
