# Supplementary material for: Core clinical competency indicators for master of nursing specialist (anaesthesia) students in China: a delphi study
Source: BMC Nurs. 2026 Jul 15;25:947. doi: 10.1186/s12912-026-05018-8 (PMC13651016; doi:10.1186/s12912-026-05018-8)
Supplement: Supplementary file 2 [file 12912_2026_5018_MOESM2_ESM.docx]

Additional file 4

The specific content of the interview outline

| Serial Number | Questions |
| --- | --- |
| 1 | Based on the job competency of anesthesia nurses, what core competencies do you believe undergraduate students in anesthesia nursing should possess? |
| 2 | How would you prioritize these core competencies in terms of their importance? |
| 3 | How do you think these core competencies are concretely demonstrated in actual work? |
| 4 | What is your opinion on the current quality of undergraduate anesthesia nursing education? Please share your views. |
| 5 | Do you have any additional comments regarding this interview? |
