# Supplementary material for: Core clinical competency indicators for master of nursing specialist (anaesthesia) students in China: a delphi study
Source: BMC Nurs. 2026 Jul 15;25:947. doi: 10.1186/s12912-026-05018-8 (PMC13651016; doi:10.1186/s12912-026-05018-8)
Supplement: Supplementary file 3 [file 12912_2026_5018_MOESM3_ESM.docx]

**Additional file 3：Delphi Study-Survey Round 1**

**Construction of the Evaluation Index System for Core Clinical Competence of Chinese Master of Nursing Specialist Postgraduates in Anesthesia (the first round)**

Dear Expert,

We are conducting a study entitled ***Construction of the Evaluation Index System for Core Clinical Competence of*** *Master of Nursing Specialist Postgraduates in Anesthesia*. The aim of this study is to establish a scientific, reasonable and domestically applicable evaluation index system for the core clinical competence of Chinese Master of Nursing Specialist Postgraduates in Anesthesia, so as to provide a reference for the training of such postgraduates. Based on previous literature research and qualitative interviews, our research group has initially constructed the evaluation index system and developed this expert consultation questionnaire. You are well recognized for your profound knowledge and rich professional experience in the fields of nursing education, clinical anesthesia nursing, anesthesia nursing management, and/or clinical anesthesiology. We sincerely invite you to serve as an expert consultant for this study. Your valuable opinions and suggestions will serve as an important basis for our research.

## General Instructions

It will take you approximately 30 to 60 minutes to finish this questionnaire. Your participation is voluntary, and you may withdraw from this study at any time. We solemnly promise that all survey data will only be used for academic research, and all information will be kept strictly anonymous and confidential throughout the study.By selecting "I consent" below, you confirm that you have fully read and understood all relevant information of this study. Please complete this round of expert consultation questionnaire within two weeks upon receipt.

We greatly appreciate your support. Please feel free to contact us if you have any questions.

I agree to participate in and complete this questionnaire.

☐ I consent

☐ I do not consent

| Contact Person： | Xueyan Xing |
| --- | --- |
| Telephone： | XXX |
| E-mail： | xingxueyan_2006@163.com |
| Affiliation： | Beijing Tsinghua Changgung Hospital, School of Clinical Medicine, Tsinghua University |

***Questionnaire Contents***

***Part 1: Evaluation Indicator Consultation Form (Table 1–3)***

***Part 2: Expert Basic Information (Table 4–6)***

**Part 1**

**The Evaluation Index System for Core Clinical Competence of Chinese Master of Nursing Specialist Postgraduates in Anesthesia**

## Instructions for Filling in the Form

Table 1, Table 2 and Table 3 list the primary, secondary and tertiary indicators of core clinical competence for **Master of Nursing Specialist postgraduates in anesthesia**.All indicators are rated by importance on a five-point scale:very important, relatively important, Moderately Important, Slightly Important, totally unimportant, corresponding to scores of 5, 4, 3, 2 and 1 respectively.Please mark a check “√” in the appropriate column.If you consider the item description inaccurate, recommend deletion, or have other comments, please revise it in the Suggestions **for Revision** column.If you think there are any unconsidered indicators that need to be added, please supplement them in the **Indicators to Be Supplemented** column. For any revised or newly added items, please also evaluate their importance and mark “√” in the corresponding column.

**Table 1 The primary indicators questionnaire**

| **Primary indicators** | Expert Comments | | | | | |
| --- | --- | --- | --- | --- | --- | --- |
|  | very important | relatively important | Moderately important | Slightly important | totally unimportant | **Your revised opinions** |
|  | **5** | **4** | **3** | **2** | **1** |  |
| 1.Pre-anesthesia health assessment |  |  |  |  |  |  |
| 2.Clinical anesthesia nursing management |  |  |  |  |  |  |
| 3. Anesthesia Monitoring |  |  |  |  |  |  |
| 4.instruments and equipment |  |  |  |  |  |  |
| 5.Termination of anesthesia |  |  |  |  |  |  |
| 6.Postoperative nursing and pain management |  |  |  |  |  |  |
| 7.Infection and control |  |  |  |  |  |  |
| 8.Anesthesia specialty documentation |  |  |  |  |  |  |
| 9.Risk management |  |  |  |  |  |  |
| 10.Clinical nursing Research |  |  |  |  |  |  |
| Supplementary Indicators |  |  |  |  |  |  |
|  |  |  |  |  |  |  |

**Table 2	The secondary indicators questionnaire**

| **Primary indicators** | **Secondary indicators** | Expert Comments | | | | | |
| --- | --- | --- | --- | --- | --- | --- | --- |
|  |  | very important | relatively important | Moderately important | Slightly important | totally unimportant | **Your revised opinions** |
|  |  | **5** | **4** | **3** | **2** | **1** |  |
| 1.Pre-anesthesia health assessment | 1.1 Comprehensive medical history assessment |  |  |  |  |  |  |
|  | 1.2 Pre-anesthesia nursing plan |  |  |  |  |  |  |
| Supplementary Indicators |  |  |  |  |  |  |  |
|  |  |  |  |  |  |  |  |
| 2.Clinical anesthesia nursing management | 2.1 Pre-anesthesia preparation |  |  |  |  |  |  |
|  | 2.2 Peri-anesthetic Medications |  |  |  |  |  |  |
|  | 2.3 Airway management |  |  |  |  |  |  |
|  | 2.4 Various Anesthesia Methods |  |  |  |  |  |  |
|  | 2.5 Anesthesia nursing for various specialist surgeries |  |  |  |  |  |  |
|  | 2.6 Anesthetic Crisis Recognition and Management |  |  |  |  |  |  |
| Supplementary Indicators |  |  |  |  |  |  |  |
|  |  |  |  |  |  |  |  |
| 3. Anesthesia Monitoring | 1. 2. 3.   3.1 ECG and Hemodynamic Monitoring |  |  |  |  |  |  |
|  | 3.2 Respiratory Gas Exchange Monitoring |  |  |  |  |  |  |
|  | 3.3 Neurological monitoring |  |  |  |  |  |  |
|  | 3.4 Other Monitoring |  |  |  |  |  |  |
| Supplementary Indicators |  |  |  |  |  |  |  |
|  |  |  |  |  |  |  |  |
| 4.instruments and equipment | 1. 2. 3. 4.   4.1 Anesthesia machine |  |  |  |  |  |  |
|  | 4.2 Ventilator |  |  |  |  |  |  |
|  | 4.3 Monitoring instruments |  |  |  |  |  |  |
|  | 4.4 Endotracheal intubation auxiliary equipment |  |  |  |  |  |  |
|  | 4.5 Other special equipment |  |  |  |  |  |  |
| Supplementary Indicators |  |  |  |  |  |  |  |
|  |  |  |  |  |  |  |  |
| 5.Termination of anesthesia | 5.1 Safe transfer of post-anesthesia patients |  |  |  |  |  |  |
|  | 5.2 Handover of post-anesthesia patients |  |  |  |  |  |  |
| Supplementary Indicators |  |  |  |  |  |  |  |
|  |  |  |  |  |  |  |  |
| 6.Postoperative nursing and pain management | 1. 2. 3. 4. 5. 6.   6.1 Post-Anesthesia Recovery Nursing |  |  |  |  |  |  |
|  | 6.2 Pain Management |  |  |  |  |  |  |
| Supplementary Indicators |  |  |  |  |  |  |  |
|  |  |  |  |  |  |  |  |
| 7.Infection and control | 7. 8. 9. 10. 11. 12.   7.1 Personal protection |  |  |  |  |  |  |
|  | 7.2 Disinfection and isolation |  |  |  |  |  |  |
|  | 7.3 Aseptic technique |  |  |  |  |  |  |
| Supplementary Indicators |  |  |  |  |  |  |  |
|  |  |  |  |  |  |  |  |
| 8.Anesthesia specialty documentation | 8.1 Anesthesia Records and PACU records |  |  |  |  |  |  |
|  | 8.2 Other documentation |  |  |  |  |  |  |
| Supplementary Indicators |  |  |  |  |  |  |  |
|  |  |  |  |  |  |  |  |
| 9.Risk management | 13. 14. 15. 16. 17. 18.   9.1 Surgical safety check |  |  |  |  |  |  |
|  | 9.2 Anesthesia quality management |  |  |  |  |  |  |
|  | 9.3 Anesthesia nursing quality management |  |  |  |  |  |  |
|  | 9.4 Adverse nursing events |  |  |  |  |  |  |
|  | 9.5 Management of Narcotic Drugs |  |  |  |  |  |  |
| Supplementary Indicators |  |  |  |  |  |  |  |
|  |  |  |  |  |  |  |  |
| 10.Clinical nursing Research | 10.1 Evidence-based clinical translation |  |  |  |  |  |  |
|  | 10.2 Clinical Research Ability |  |  |  |  |  |  |
| Supplementary Indicators |  |  |  |  |  |  |  |
|  |  |  |  |  |  |  |  |

**Table 3 The tertiary indicators questionnaire**

| **Primary indicators** | **Secondary indicators** | **Tertiary indicators** | Expert Comments | | | | | |
| --- | --- | --- | --- | --- | --- | --- | --- | --- |
|  |  |  | very important | relatively important | Moderately important | Slightly important | totally unimportant | **Your**  **revised opinions** |
|  |  |  | **5** | **4** | **3** | **2** | **1** |  |
| 1.Pre-anesthesia health assessment | 1.1 Comprehensive medical history assessment | 1.1.1 Know the patient's height, weight and vital signs |  |  |  |  |  |  |
|  |  | 1.1.2 Know positive signs of physical examination |  |  |  |  |  |  |
|  |  | 1.1.3 Know the patient's current medical history |  |  |  |  |  |  |
|  |  | 1.1.4 Know the patient's past medical history |  |  |  |  |  |  |
|  |  | 1.1.5 Know the positive indicators in the results of imaging examinations and laboratory tests |  |  |  |  |  |  |
|  |  | 1.1.6 Know the assessment of preoperative therapeutic medications |  |  |  |  |  |  |
|  |  | 1.1.7 Familiar with ASA classification |  |  |  |  |  |  |
|  | 1.2 Pre-anesthesia nursing plan | 1.2.1 Possess the ability to formulate an anesthesia nursing plan based on Pre-anesthesia nursing assessment results, combined with current knowledge, concepts, available evidence, and nursing principles |  |  |  |  |  |  |
|  |  | 1.2.2 Assessing patients’ psychological status and providing timely psychological care |  |  |  |  |  |  |
| Supplementary Indicators |  |  |  |  |  |  |  |  |
|  |  |  |  |  |  |  |  |  |
| 2.Clinical anesthesia nursing management | 2.1 Pre-anesthesia preparation | 2.1.1 Master the preoperative medication preparation for various anesthesia methods |  |  |  |  |  |  |
|  |  | 2.1.2 Master the preparation of preoperative supplies for various anesthesia methods |  |  |  |  |  |  |
|  | 2.2 Peri-anesthetic Medications | 2.2.1 Familiar with the pharmacological effects, dosage, administration methods and adverse reactions of various anesthetic drugs |  |  |  |  |  |  |
|  |  | 2.2.2 Familiar with the pharmacological effects, dosage, administration methods and adverse reactions of various analgesic drugs |  |  |  |  |  |  |
|  |  | 2.2.3 Familiar with the pharmacological effects, dosage, administration methods and adverse reactions of various muscle relaxants |  |  |  |  |  |  |
|  |  | 2.2.4 Familiar with the pharmacological effects, dosage, administration methods and adverse reactions of various first-aid drugs |  |  |  |  |  |  |
|  |  | 2.2.5 Familiar with the pharmacological effects, dosage, administration methods and adverse reactions of other perioperative adjuvant drugs |  |  |  |  |  |  |
|  | 2.3 Airway management | 2.3.1 Master mask ventilation techniques, including EC clamp technique and two-hand mask ventilation technique |  |  |  |  |  |  |
|  |  | 2.3.2 Master the insertion techniques of oropharyngeal airways and nasopharyngeal airways |  |  |  |  |  |  |
|  |  | 2.3.3 Familiar with the structure of supraglottic airway devices and their insertion operation procedures, and be able to assist in completing the insertion |  |  |  |  |  |  |
|  |  | 2.3.4 Familiar with the structure and insertion procedures of various endotracheal tubes and bronchial tubes, and be able to assist in completing the insertion |  |  |  |  |  |  |
|  |  | 2.3.5 Familiar with the difficult airway management process |  |  |  |  |  |  |
|  | 2.4 Various Anesthesia Methods | 2.4.1 Master the principles, complications, and processes of general anesthesia, and be able to assist anesthesiologists in performing general anesthesia |  |  |  |  |  |  |
|  |  | 2.4.2 Master the principles and complications of neuraxial anesthesia, and be able to assist anesthesiologists in performing neuraxial anesthesia |  |  |  |  |  |  |
|  |  | 2.4.3 Master the principles and complications of peripheral nerve block, and be able to assist anesthesiologists in performing peripheral nerve block |  |  |  |  |  |  |
|  | 2.5 Anesthesia nursing for various specialist surgeries | 2.5.1 Familiar with the characteristics of surgeries in various specialties and their impacts on anesthesia |  |  |  |  |  |  |
|  |  | 2.5.2 Familiar with anesthetic management of common surgeries across various specialties |  |  |  |  |  |  |
|  |  | 2.5.3 Be able to assist in correctly positioning surgical patients, maintain unobstructed pipelines, and protect compressed skin areas and nerve plexuses. |  |  |  |  |  |  |
|  |  | 2.5.4 Possess the ability to comprehensively evaluate the patient's condition based on various monitoring indicators, identify potential risks endangering patient safety, and implement appropriate countermeasures |  |  |  |  |  |  |
|  |  | 2.5.5 Be able to conduct dynamic assessment and adjustment based on various data while implementing the anesthesia nursing plan |  |  |  |  |  |  |
|  |  | 2.5.6 Be able to employ appropriate communication skills, provide psychological support, and help patients get through the perioperative period |  |  |  |  |  |  |
|  | 2.6 Anesthetic Crisis Recognition and Management | 2.6.1 Be able to rapidly assess emergent critical incidents, deliver urgent interventions within the scope of professional responsibilities, and activate emergency protocols when necessary |  |  |  |  |  |  |
|  |  | 2.6.2 Be able to promptly seek assistance from operating room nurses, anesthesiologists, surgeons, and other staff, and actively cooperate with the team in emergency treatment |  |  |  |  |  |  |
| Supplementary Indicators |  |  |  |  |  |  |  |  |
|  |  |  |  |  |  |  |  |  |
| 3. Anesthesia Monitoring | 4. 5. 6.   3.1 ECG and Hemodynamic Monitoring | 3.1.1 Master the monitoring methods of electrocardiography (ECG), grasp the characteristics of normal ECG, and be familiar with the ECG manifestations of common arrhythmias |  |  |  |  |  |  |
|  |  | 3.1.2 Master the monitoring methods and normal reference ranges for common arterial and venous pressure parameters (including NBP, ABP, CVP, etc.) |  |  |  |  |  |  |
|  |  | 3.1.3 Master the principles and methods of pulmonary artery pressure monitoring |  |  |  |  |  |  |
|  |  | 3.1.4 Master invasive monitoring techniques for cardiac output |  |  |  |  |  |  |
|  | 3.2 Respiratory Gas Exchange Monitoring | 3.2.1 Familiar with the monitoring and normal reference ranges of respiratory parameters (tidal volume, respiratory rate, airway pressure, end-tidal carbon dioxide, etc.) |  |  |  |  |  |  |
|  |  | 3.2.2 Master the monitoring method and normal reference range of pulse oximetry saturation |  |  |  |  |  |  |
|  | 3.3 Neurological monitoring | 3.3.1 Master the monitoring methods and normal reference ranges of Bispectral Index (BIS) |  |  |  |  |  |  |
|  |  | 3.3.2 Familiar with the monitoring methods and normal reference ranges of regional cerebral oxygen saturation (rSO₂) |  |  |  |  |  |  |
|  | 3.4 Other Monitoring | 3.4.1 Master the methods and normal reference ranges of perioperative temperature monitoring |  |  |  |  |  |  |
|  |  | 3.4.2 Master the methods of maintaining normal body temperature in perioperative care |  |  |  |  |  |  |
|  |  | 3.4.3 Master urine output monitoring |  |  |  |  |  |  |
|  |  | 3.4.4 Know monitoring methods of neuromuscular function |  |  |  |  |  |  |
| Supplementary Indicators |  |  |  |  |  |  |  |  |
|  |  |  |  |  |  |  |  |  |
| 4.instruments and equipment | 5. 6. 7. 8.   4.1 Anesthesia machine | 4.1.1 Master the self-check and routine operation of anesthesia machines, including analysis and management of common alarms |  |  |  |  |  |  |
|  |  | 4.1.2 Know the causes and management of common faults of anesthesia machines |  |  |  |  |  |  |
|  | 4.2 Ventilator | 4.2.1 Master the self-check and routine operation of ventilators, including the analysis and management of common alarms |  |  |  |  |  |  |
|  |  | 4.2.2 Know the causes and management of common ventilator malfunctions |  |  |  |  |  |  |
|  | 4.3 Monitoring instruments | 4.3.1 Master the correct operating procedures for multi-parameter monitors ，including analysis and management of various alarms |  |  |  |  |  |  |
|  |  | 4.3.2 Familiar with the standard operating procedures for cardiac output monitors |  |  |  |  |  |  |
|  |  | 4.3.3 Master the standard operating procedures of BIS monitors |  |  |  |  |  |  |
|  |  | 4.3.4 Familiar with the standard operating procedures of cerebral oximetry monitors |  |  |  |  |  |  |
|  |  | 4.3.5 Know the correct operating procedures for neuromuscular monitoring devices |  |  |  |  |  |  |
|  | 4.4 Endotracheal intubation auxiliary equipment | 4.4.1 Master the structure of conventional laryngoscopes and the correct methods for assembling and disassembling blades. |  |  |  |  |  |  |
|  |  | 4.4.2 Master the structure of common video laryngoscopes and the correct methods for installing and removing blades |  |  |  |  |  |  |
|  |  | 4.4.3 Master the structure of fiberoptic bronchoscopes |  |  |  |  |  |  |
|  | 4.5 Other special equipment | 4.5.1 Master the correct operation and troubleshooting methods of syringe pumps and infusion pumps |  |  |  |  |  |  |
|  |  | 4.5.2 Master the correct operating methods and troubleshooting techniques for analgesia pumps |  |  |  |  |  |  |
|  |  | 4.5.3 Master the correct operation and troubleshooting of conductive heating devices (e.g., circulating water blankets, electric heating blankets) |  |  |  |  |  |  |
|  |  | 4.5.4 Master the correct operating procedures and troubleshooting techniques for forced-air warming devices |  |  |  |  |  |  |
|  |  | 4.5.5 Master the correct operating procedures and troubleshooting methods for fluid warming devices |  |  |  |  |  |  |
|  |  | 4.5.6 Know the structure and application of blood salvage equipment |  |  |  |  |  |  |
|  |  | 4.5.7 Know the structure and operation of extracorporeal circulation equipment |  |  |  |  |  |  |
| Supplementary Indicators |  |  |  |  |  |  |  |  |
|  |  |  |  |  |  |  |  |  |
| 5.Termination of anesthesia | 1. 2. 3. 4. 5. 1. 2. 3. 4. 5.   5.1 Safe transfer of post-anesthesia patients | 5.1.1 Master the pre-transfer assessment of postoperative patients |  |  |  |  |  |  |
|  |  | 5.1.2 Master all preparations (including medications, supplies, instruments and equipment) prior to the transfer of postoperative patients |  |  |  |  |  |  |
|  |  | 5.1.3 Familiar with the procedures and key focuses for the transfer of postoperative patients |  |  |  |  |  |  |
|  | 5.2 Handover of post-anesthesia patients | 5.2.1 Master the handover content with nurses from the PACU, ICU, and general wards |  |  |  |  |  |  |
| Supplementary Indicators |  |  |  |  |  |  |  |  |
|  |  |  |  |  |  |  |  |  |
| 6.Postoperative nursing and pain management | 19. 20. 21. 22. 23. 24.   6.1 Post-Anesthesia Recovery Nursing | 6.1.1 Master the management regulations and routine work protocols of the PACU |  |  |  |  |  |  |
|  |  | 6.1.2 Master the post-anesthesia nursing characteristics of various specialized surgeries, as well as the assessment and nursing care of common anesthetic complications |  |  |  |  |  |  |
|  |  | 6.1.3Master the postoperative nursing processes, as well as the assessment and nursing of complications for various specialty surgeries |  |  |  |  |  |  |
|  | 6.2 Pain Management | 6.2.1 Know the principles of perioperative pain management |  |  |  |  |  |  |
|  |  | 6.2.2 Master commonly used pain assessment methods |  |  |  |  |  |  |
|  |  | 6.2.3 Master nursing care for surgical patients' pain, assist in implementing pain interventions, and promptly manage complications related to analgesic therapy |  |  |  |  |  |  |
|  |  | 6.2.4 Master the follow-up content and workflow of postoperative analgesia |  |  |  |  |  |  |
| Supplementary Indicators |  |  |  |  |  |  |  |  |
|  |  |  |  |  |  |  |  |  |
| 7.Infection and control | 25. 26. 27. 28. 29. 30.   7.1 Personal protection | 7.1.1 Be able to select appropriate cleaning and disinfection devices and perform correct hand hygiene practices |  |  |  |  |  |  |
|  |  | 7.1.2 Be able to implement standard precautions correctly according to specific clinical situations |  |  |  |  |  |  |
|  | 7.2 Disinfection and isolation | 7.2.1 Know the disinfection management of commonly used instruments and supplies in the anesthesiology department |  |  |  |  |  |  |
|  |  | 7.2.2 Be able to properly dispose of medical waste, including sharps |  |  |  |  |  |  |
|  | 7.3 Aseptic technique | 7.3.1 Master aseptic principles |  |  |  |  |  |  |
|  |  | 7.3.2 Master commonly used aseptic practice methods |  |  |  |  |  |  |
| Supplementary Indicators |  |  |  |  |  |  |  |  |
|  |  |  |  |  |  |  |  |  |
| 8.Anesthesia specialty documentation | 8.1 Anesthesia Records and PACU records | 8.1.1 Be able to complete anesthesia records in a timely, comprehensive and accurate manner. |  |  |  |  |  |  |
|  |  | 8.1.2 Be able to complete PACU records in a timely, comprehensive and accurate manner |  |  |  |  |  |  |
|  | 8.2 Other documentation | 8.2.1 Be able to fill out postoperative handover forms completely and accurately |  |  |  |  |  |  |
|  |  | 8.2.2 Be able to document postoperative analgesia follow‑up records in a timely, complete and accurate manner |  |  |  |  |  |  |
| Supplementary Indicators |  |  |  |  |  |  |  |  |
|  |  |  |  |  |  |  |  |  |
| 9.Risk management | 31. 32. 33. 34. 35. 36.   9.1 Surgical safety check | 9.1.1 Familiar with the timing of surgical safety checks |  |  |  |  |  |  |
|  |  | 9.1.2 Know the content of surgical safety checks |  |  |  |  |  |  |
|  | 9.2 Anesthesia quality management | 9.2.1 Know the types and definitions of anesthesia quality indicators |  |  |  |  |  |  |
|  |  | 9.2.2 Know the thresholds of various anesthesia quality indicators |  |  |  |  |  |  |
|  | 9.3 Anesthesia nursing quality management | 9.3.1 Know rules and regulations related to anesthesia nursing |  |  |  |  |  |  |
|  |  | 9.3.2 Familiar with standard operating procedures for anesthesia nursing |  |  |  |  |  |  |
|  |  | 9.3.3 Master the categories and definitions of quality control indicators for anesthesia nursing |  |  |  |  |  |  |
|  |  | 9.3.4 Know common quality management methods |  |  |  |  |  |  |
|  | 9.4 Adverse nursing events | 9.4.1 Master the classification of adverse nursing events |  |  |  |  |  |  |
|  |  | 9.4.2 Master risk assessment for various adverse nursing events |  |  |  |  |  |  |
|  |  | 9.4.3 Master preventive measures for all types of adverse nursing events |  |  |  |  |  |  |
|  |  | 9.4.4 Master the handling and reporting procedures of adverse nursing events |  |  |  |  |  |  |
|  | 9.5 Management of Narcotic Drugs | 9.5.1 Master the classification and management requirements of narcotic and psychotropic drugs, including storage, requisition, administration and return procedures |  |  |  |  |  |  |
|  |  | 9.5.2 Master the classification and management requirements of other high-risk medications in the anesthesiology department, including storage, requisition, administration, and return procedures |  |  |  |  |  |  |
| Supplementary Indicators |  |  |  |  |  |  |  |  |
|  |  |  |  |  |  |  |  |  |
| 10.Clinical nursing Research | 10.1 Evidence-based clinical translation | 10.1.1 Be able to formulate appropriate search strategies for evidence retrieval |  |  |  |  |  |  |
|  |  | 10.1.2 Be able to conduct comprehensive literature evaluation and provide valuable evidence-based references |  |  |  |  |  |  |
|  |  | 10.1.3 Be able to apply valid evidence in anesthesia nursing practice |  |  |  |  |  |  |
|  |  | 10.1.4 Be able to put forward suggestions for formulating evidence‑based nursing practice protocols and quality standards after evaluating the application scenarios of evidence |  |  |  |  |  |  |
|  | 10.2 Clinical Research Ability | 10.2.1 Possess the ability to actively transform clinical problems into research questions |  |  |  |  |  |  |
|  |  | 10.2.2 Be able to select appropriate research methods and statistical approaches for research design, conduct clinical studies, generate research outcomes, and propose recommendations for further investigations |  |  |  |  |  |  |
| Supplementary Indicators |  |  |  |  |  |  |  |  |
|  |  |  |  |  |  |  |  |  |

**Part 2 Expert Basic Information**

## 【Instructions】

This survey is only used for the present research project. All information provided will be kept strictly confidential by the research team. Please fill in the form according to your actual personal situation, or tick “√” beside the appropriate option. If further explanation is needed, please make remarks in the corresponding column.

**1.Personal Information**

| **Table 4 Expert Basic Information Sheet** | | | | | | | |
| --- | --- | --- | --- | --- | --- | --- | --- |
| Age： |  | | | Gender： | □ Male | | □Female |
| Work unit and department： |  | | | Administrative position： |  | | |
| Education / Degree： | □Doctoral | | □Master | | | □Bachelor | |
| Professional title： | □Senior Professor | | □Associate Senior Professor | | | □Intermediate | |
| Main work field： | □Clinical Anesthesiology | | □Clinical Anesthesia Nursing | | | | |
|  | □Nursing Education | | □Anesthesia Nursing Management | | | | |
|  | □Others (please specify)： |  | | | | | |
| Years of working in the above field(s)： | years | | | | | | |
| Are you a supervisor? | □NO | | □Master’s Supervisor (Professional Degree) | | | | |
|  | □Doctoral Supervisor | | □Master’s Supervisor (Academic Degree) | | | | |
| Preferred method for next consultation： | □E-mail: | | | | | | |
|  | □WeChat: | | | | | | |

# 2.Experts' Familiarity with the Consultation Content and Basis for Judgment

| Table 5 Experts' Familiarity with the Consultation Content | | | | | |
| --- | --- | --- | --- | --- | --- |
| Degree of familiarity | Most Familiar | More familiar | Familiar | Less familiar | Unfamiliar |
| Expert self-evaluation |  |  |  |  |  |

| Table 6 Experts' Basis for Judgment | | | | | |
| --- | --- | --- | --- | --- | --- |
| Basis for making judgments | The extent of the impact on the significance of the indicators | | | | |
|  | Significant | | intermediate | Insignificance | |
| Theoretical Analysis |  |  | | |  |
| Practical Experience |  |  | | |  |
| reference documentation |  |  | | |  |
| Intuitive Judgment |  |  | | |  |
