# Supplementary material for: Core clinical competency indicators for master of nursing specialist (anaesthesia) students in China: a delphi study
Source: BMC Nurs. 2026 Jul 15;25:947. doi: 10.1186/s12912-026-05018-8 (PMC13651016; doi:10.1186/s12912-026-05018-8)
Supplement: Supplementary file 4 [file 12912_2026_5018_MOESM4_ESM.docx]

**Additional file 4：Delphi Study-Survey Round 2**

**Construction of the Evaluation Index System for Core Clinical Competence of Chinese Master of Nursing Specialist Postgraduates in Anesthesia (the second round)**

Dear Expert,

Thank you for participating in this study and completing the first round of consultation. The theme of this study is **Construction of the Evaluation Index System for Core Clinical Competence of** Master of Nursing Specialist Postgraduates in Anesthesia.This study aims to construct a scientific, reasonable and domestically applicable evaluation index system for the core clinical competence of Chinese Master of Nursing Specialist Postgraduates in Anesthesia, so as to provide a reference for the training of Master of Nursing Specialist Postgraduates in Anesthesia.You are acknowledged to have profound knowledge and rich experience in the fields of nursing education, clinical anesthesia nursing, anesthesia nursing management, and/or clinical anesthesiology practice. We sincerely invite you to complete this expert consultation questionnaire based on your professional knowledge and work experience. Your valuable opinions and suggestions will serve as an important basis for this research project.

## General Instructions

It will take you approximately 30 to 60 minutes to finish this questionnaire. Your participation is voluntary, and you may withdraw from this study at any time. We solemnly promise that all survey data will only be used for academic research, and all information will be kept strictly anonymous and confidential throughout the study.By selecting "I consent" below, you confirm that you have fully read and understood all relevant information of this study. Please complete the second round of expert consultation questionnaire within two weeks upon receipt.

We greatly appreciate your support. Please feel free to contact us if you have any questions.

I agree to participate in and complete this questionnaire.

☐ I consent

☐ I do not consent

| Contact Person： | Xueyan Xing |
| --- | --- |
| Telephone： | XXX |
| E-mail： | xingxueyan_2006@163.com |
| Affiliation： | Beijing Tsinghua Changgung Hospital, School of Clinical Medicine, Tsinghua University |

**The Evaluation Index System for Core Clinical Competence of Chinese Master of Nursing Specialist Postgraduates in Anesthesia**

## Instructions for Filling in the Form

Table 1, Table 2 and Table 3 list the primary, secondary and tertiary indicators of core clinical competence for **Master of Nursing Specialist postgraduates in anesthesia**.All indicators are rated by importance on a five-point scale:very important, relatively important, Moderately Important, Slightly Important, totally unimportant, corresponding to scores of 5, 4, 3, 2 and 1 respectively.Please mark a check “√” in the appropriate column.If you consider the item description inaccurate, recommend deletion, or have other comments, please revise it in the Suggestions **for Revision** column.If you think there are any unconsidered indicators that need to be added, please supplement them in the **Indicators to Be Supplemented** column. For any revised or newly added items, please also evaluate their importance and mark “√” in the corresponding column.

Should you have any questions while completing the form, please feel free to contact us at any time.

**Table 1 The primary indicators questionnaire**

| **Primary indicators** | Expert Comments | | | | | |
| --- | --- | --- | --- | --- | --- | --- |
|  | very important | relatively important | Moderately important | Slightly important | totally unimportant | **Your revised opinions** |
|  | **5** | **4** | **3** | **2** | **1** |  |
| 1. Pre-anesthesia nursing assessment |  |  |  |  |  |  |
| 2. Clinical anesthesia nursing |  |  |  |  |  |  |
| 3. Anesthesia Monitoring |  |  |  |  |  |  |
| 4. Application of instruments and equipment |  |  |  |  |  |  |
| 5. Infection prevention and control |  |  |  |  |  |  |
| 6. Anesthesia documentation |  |  |  |  |  |  |
| 7. Risk management |  |  |  |  |  |  |
| 8. Clinical nursing Research |  |  |  |  |  |  |
| Supplementary Indicators |  |  |  |  |  |  |
|  |  |  |  |  |  |  |

**Table 2	The secondary indicators questionnaire**

| **Primary indicators** | **Secondary indicators** | Expert Comments | | | | | |
| --- | --- | --- | --- | --- | --- | --- | --- |
|  |  | very important | relatively important | Moderately important | Slightly important | totally unimportant | **Your revised opinions** |
|  |  | **5** | **4** | **3** | **2** | **1** |  |
| 1. Pre-anesthesia nursing assessment | 1.1 General information assessment |  |  |  |  |  |  |
|  | 1.2 Physical status assessment |  |  |  |  |  |  |
|  | 1.3 Comprehensive medical history assessment |  |  |  |  |  |  |
|  | 1.4 Risk assessment |  |  |  |  |  |  |
|  | 1.5 Pre-anesthesia nursing plan |  |  |  |  |  |  |
| Supplementary Indicators |  |  |  |  |  |  |  |
|  |  |  |  |  |  |  |  |
| 2. Clinical anesthesia nursing | 2.1 Pre-anesthesia preparation |  |  |  |  |  |  |
|  | 2.2 Knowledge of drugs during the Peri-anesthesia period |  |  |  |  |  |  |
|  | 2.3 Airway management |  |  |  |  |  |  |
|  | 2.4 Assistance with various anesthesia methods |  |  |  |  |  |  |
|  | 2.5 Anesthesia nursing for various specialist surgeries |  |  |  |  |  |  |
|  | 2.6 Anesthetic Crisis Recognition and Management |  |  |  |  |  |  |
|  | 1. 2. 3. 4. 5. 1. 2. 3. 4. 5.   2.7 Safe transfer and handover of postoperative patients |  |  |  |  |  |  |
|  | 2.8 Monitoring and nursing in the PACU |  |  |  |  |  |  |
|  | 2.9 Pain Management |  |  |  |  |  |  |
| Supplementary Indicators |  |  |  |  |  |  |  |
|  |  |  |  |  |  |  |  |
| 3. Anesthesia Monitoring | 1. 2. 3.   3.1 Circulatory system monitoring |  |  |  |  |  |  |
|  | 3.2 Respiratory function monitoring |  |  |  |  |  |  |
|  | 3.3 Neurological monitoring |  |  |  |  |  |  |
|  | 3.4 Temperature monitoring |  |  |  |  |  |  |
|  | 3.5 Intake and output monitoring |  |  |  |  |  |  |
|  | 3.6 Neuromuscular transmission function monitoring |  |  |  |  |  |  |
| Supplementary Indicators |  |  |  |  |  |  |  |
|  |  |  |  |  |  |  |  |
| 4. Application of instruments and equipment | 1. 2. 3. 4.   4.1 Anesthesia machine |  |  |  |  |  |  |
|  | 4.2 Ventilator |  |  |  |  |  |  |
|  | 4.3 Monitoring instruments |  |  |  |  |  |  |
|  | 4.4 Endotracheal intubation auxiliary equipment |  |  |  |  |  |  |
|  | 4.5 Medical infusion devices |  |  |  |  |  |  |
|  | 4.6 Perioperative warming devices |  |  |  |  |  |  |
|  | 4.7 Other special equipment |  |  |  |  |  |  |
| Supplementary Indicators |  |  |  |  |  |  |  |
|  |  |  |  |  |  |  |  |
| 5. Infection prevention and control | 1. 2. 3. 4. 5. 6.   5.1 Personal protection |  |  |  |  |  |  |
|  | 5.2 Disinfection and isolation |  |  |  |  |  |  |
|  | 5.3 Aseptic technique |  |  |  |  |  |  |
| Supplementary Indicators |  |  |  |  |  |  |  |
|  |  |  |  |  |  |  |  |
| 6. Anesthesia documentation | 6.1 Anesthesia Records and PACU records |  |  |  |  |  |  |
|  | 6.2 Other documentation |  |  |  |  |  |  |
| Supplementary Indicators |  |  |  |  |  |  |  |
|  |  |  |  |  |  |  |  |
| 7. Risk management | 7. 8. 9. 10. 11. 12.   7.1 Surgical safety check |  |  |  |  |  |  |
|  | 7.2 Anesthesia quality management |  |  |  |  |  |  |
|  | 7.3 Anesthesia nursing quality management |  |  |  |  |  |  |
|  | 7.4 Adverse nursing events |  |  |  |  |  |  |
|  | 7.5 Management of high-risk medications in anesthesiology |  |  |  |  |  |  |
| Supplementary Indicators |  |  |  |  |  |  |  |
|  |  |  |  |  |  |  |  |
| 8. Clinical nursing Research | 8.1 Evidence-based clinical translation |  |  |  |  |  |  |
|  | 8.2 Original research |  |  |  |  |  |  |
| Supplementary Indicators |  |  |  |  |  |  |  |
|  |  |  |  |  |  |  |  |

**Table 3 The tertiary indicators questionnaire**

| **Primary indicators** | **Secondary indicators** | **Tertiary indicators** | Expert Comments | | | | | |
| --- | --- | --- | --- | --- | --- | --- | --- | --- |
|  |  |  | very important | relatively important | Moderately important | Slightly important | totally unimportant | **Your revised opinions** |
|  |  |  | **5** | **4** | **3** | **2** | **1** |  |
| 1. Pre-anesthesia nursing assessment | 1.1 General information assessment | 1.1.1 Know patients’ basic information, including gender, age, ethnicity, place of birth, education level and occupation |  |  |  |  |  |  |
|  |  | 1.1.2 Know patients’ privacy needs, family support and religious beliefs |  |  |  |  |  |  |
|  | 1.2 Physical status assessment | 1.2.1 Know the patient's height, weight and vital signs |  |  |  |  |  |  |
|  |  | 1.2.2 Know positive signs of physical examination |  |  |  |  |  |  |
|  | 1.3 Comprehensive medical history assessment | 1.3.1 Know the patient's current medical history |  |  |  |  |  |  |
|  |  | 1.3.2 Know the patient's past medical history |  |  |  |  |  |  |
|  |  | 1.3.3 Know the positive indicators in the results of imaging examinations and laboratory tests |  |  |  |  |  |  |
|  |  | 1.3.4 Know the assessment of preoperative therapeutic medications |  |  |  |  |  |  |
|  | 1.4 Risk assessment | 1.4.1 Familiar with ASA classification |  |  |  |  |  |  |
|  |  | 1.4.2 Possess the ability to assess patients using relevant scales, such as those for falls, pressure injuries, and frailty |  |  |  |  |  |  |
|  | 1.5 Pre-anesthesia nursing plan | 1.5.1 Possess the ability to formulate an anesthesia nursing plan based on Pre-anesthesia nursing assessment results, combined with current knowledge, concepts, available evidence, and nursing principles |  |  |  |  |  |  |
| Supplementary Indicators |  |  |  |  |  |  |  |  |
|  |  |  |  |  |  |  |  |  |
| 2. Clinical anesthesia nursing | 2.1 Pre-anesthesia preparation | 2.1.1 Master the preoperative medication preparation for various anesthesia methods |  |  |  |  |  |  |
|  |  | 2.1.2 Master the preparation of preoperative supplies for various anesthesia methods |  |  |  |  |  |  |
|  | 2.2 Knowledge of drugs during the Peri-anesthesia period | 2.2.1 Familiar with the pharmacological effects, dosage, administration methods and adverse reactions of various anesthetic drugs |  |  |  |  |  |  |
|  |  | 2.2.2 Familiar with the pharmacological effects, dosage, administration methods and adverse reactions of various analgesic drugs |  |  |  |  |  |  |
|  |  | 2.2.3 Familiar with the pharmacological effects, dosage, administration methods and adverse reactions of various muscle relaxants |  |  |  |  |  |  |
|  |  | 2.2.4 Familiar with the pharmacological effects, dosage, administration methods and adverse reactions of various first-aid drugs |  |  |  |  |  |  |
|  |  | 2.2.5 Familiar with the pharmacological effects, dosage, administration methods and adverse reactions of other perioperative adjuvant drugs |  |  |  |  |  |  |
|  | 2.3 Airway management | 2.3.1 Master mask ventilation techniques, including EC clamp technique and two-hand mask ventilation technique |  |  |  |  |  |  |
|  |  | 2.3.2 Master the insertion techniques of oropharyngeal airways and nasopharyngeal airways |  |  |  |  |  |  |
|  |  | 2.3.3 Familiar with the structure of supraglottic airway devices and their insertion operation procedures, and be able to assist in completing the insertion |  |  |  |  |  |  |
|  |  | 2.3.4 Familiar with the structure and insertion procedures of various endotracheal tubes and bronchial tubes, and be able to assist in completing the insertion |  |  |  |  |  |  |
|  |  | 2.3.5 Familiar with the difficult airway management process |  |  |  |  |  |  |
|  | 2.4 Assistance with various anesthesia methods | 2.4.1 Master the principles, complications, and processes of general anesthesia, and be able to assist anesthesiologists in performing general anesthesia |  |  |  |  |  |  |
|  |  | 2.4.2 Master the principles and complications of neuraxial anesthesia, and be able to assist anesthesiologists in performing neuraxial anesthesia |  |  |  |  |  |  |
|  |  | 2.4.3 Master the principles and complications of peripheral nerve block, and be able to assist anesthesiologists in performing peripheral nerve block |  |  |  |  |  |  |
|  | 2.5 Anesthesia nursing for various specialist surgeries | 2.5.1 Familiar with the characteristics of surgeries in various specialties and their impacts on anesthesia |  |  |  |  |  |  |
|  |  | 2.5.2 Familiar with anesthetic management of common surgeries across various specialties |  |  |  |  |  |  |
|  |  | 2.5.3 Be able to assist in correctly positioning surgical patients, maintain unobstructed pipelines, and protect compressed skin areas and nerve plexuses. |  |  |  |  |  |  |
|  |  | 2.5.4 Possess the ability to comprehensively evaluate the patient's condition based on various monitoring indicators, identify potential risks endangering patient safety, and implement appropriate countermeasures |  |  |  |  |  |  |
|  |  | 2.5.5 Be able to conduct dynamic assessment and adjustment based on various data while implementing the anesthesia nursing plan |  |  |  |  |  |  |
|  |  | 2.5.6 Be able to employ appropriate communication skills, provide psychological support, and help patients get through the perioperative period |  |  |  |  |  |  |
|  | 2.6 Anesthetic Crisis Recognition and Management | 2.6.1 Be able to rapidly assess emergent critical incidents, deliver urgent interventions within the scope of professional responsibilities, and activate emergency protocols when necessary |  |  |  |  |  |  |
|  |  | 2.6.2 Be able to promptly seek assistance from operating room nurses, anesthesiologists, surgeons, and other staff, and actively cooperate with the team in emergency treatment |  |  |  |  |  |  |
|  | 6. 7. 8. 9. 10. 6. 7. 8. 9. 10.   2.7 Safe transfer and handover of postoperative patients | 2.7.1 Master the pre-transfer assessment of postoperative patients |  |  |  |  |  |  |
|  |  | 2.7.2 Master all preparations (including medications, supplies, instruments and equipment) prior to the transfer of postoperative patients |  |  |  |  |  |  |
|  |  | 2.7.3 Familiar with the procedures and key focuses for the transfer of postoperative patients |  |  |  |  |  |  |
|  |  | 2.7.4 Familiar with emergency management of unexpected incidents during the transfer of postoperative patients |  |  |  |  |  |  |
|  |  | 2.7.5 Master the content and procedures of handover with nurses from the PACU, ICU, and general wards |  |  |  |  |  |  |
|  | 2.8 Monitoring and nursing in the PACU | 2.8.1 Master the management regulations and routine work protocols of the PACU |  |  |  |  |  |  |
|  |  | 2.8.2 Master the post-anesthesia nursing characteristics of various specialized surgeries, as well as the assessment and nursing care of common anesthetic complications |  |  |  |  |  |  |
|  |  | 2.8.3 Master the early postoperative nursing processes, key points, and the assessment and nursing of complications for various specialty surgeries |  |  |  |  |  |  |
|  |  | 2.8.4 Master risk identification and management during the anesthesia recovery period |  |  |  |  |  |  |
|  | 2.9 Pain Management | 2.9.1 Know the principles of perioperative pain management |  |  |  |  |  |  |
|  |  | 2.9.2 Master commonly used pain assessment methods |  |  |  |  |  |  |
|  |  | 2.9.3 Master nursing care for surgical patients' pain, assist in implementing pain interventions, and promptly manage complications related to analgesic therapy |  |  |  |  |  |  |
|  |  | 2.9.4 Master the follow-up content and workflow of postoperative analgesia |  |  |  |  |  |  |
| Supplementary Indicators |  |  |  |  |  |  |  |  |
|  |  |  |  |  |  |  |  |  |
| 3. Anesthesia Monitoring | 4. 5. 6.   3.1 Circulatory system monitoring | 3.1.1 Master the monitoring methods of electrocardiography (ECG), grasp the characteristics of normal ECG, and be familiar with the ECG manifestations of common arrhythmias |  |  |  |  |  |  |
|  |  | 3.1.2 Master the monitoring methods and normal reference ranges for common arterial and venous pressure parameters (including NBP, ABP, CVP, etc.) |  |  |  |  |  |  |
|  |  | 3.1.3 Master the principles and methods of pulmonary artery pressure monitoring |  |  |  |  |  |  |
|  |  | 3.1.4 Master invasive monitoring techniques for cardiac output |  |  |  |  |  |  |
|  | 3.2 Respiratory function monitoring | 3.2.1 Familiar with the monitoring and normal reference ranges of respiratory parameters (tidal volume, respiratory rate, airway pressure, end-tidal carbon dioxide, etc.) |  |  |  |  |  |  |
|  |  | 3.2.2 Master the monitoring method and normal reference range of pulse oximetry saturation |  |  |  |  |  |  |
|  | 3.3 Neurological monitoring | 3.3.1 Master the monitoring methods and normal reference ranges of Bispectral Index (BIS) |  |  |  |  |  |  |
|  |  | 3.3.2 Familiar with the monitoring methods and normal reference ranges of regional cerebral oxygen saturation (rSO₂) |  |  |  |  |  |  |
|  | 3.4 Temperature monitoring | 3.4.1 Master the methods and normal reference ranges of perioperative temperature monitoring |  |  |  |  |  |  |
|  |  | 3.4.2 Master the methods of maintaining normal body temperature in perioperative care |  |  |  |  |  |  |
|  | 3.5 Intake and output monitoring | 3.5.1 Master the content and methods of intake monitoring |  |  |  |  |  |  |
|  |  | 3.5.2 Master the content and methods of output monitoring |  |  |  |  |  |  |
|  | 3.6 Neuromuscular transmission function monitoring | 3.6.1 Know monitoring methods of neuromuscular function |  |  |  |  |  |  |
| Supplementary Indicators |  |  |  |  |  |  |  |  |
|  |  |  |  |  |  |  |  |  |
| 4. Application of instruments and equipment | 5. 6. 7. 8.   4.1 Anesthesia machine | 4.1.1 Master the self-check and routine operation of anesthesia machines, including analysis and management of common alarms |  |  |  |  |  |  |
|  |  | 4.1.2 Know the causes and management of common faults of anesthesia machines |  |  |  |  |  |  |
|  | 4.2 Ventilator | 4.2.1 Master the self-check and routine operation of ventilators, including the analysis and management of common alarms |  |  |  |  |  |  |
|  |  | 4.2.2 Know the causes and management of common ventilator malfunctions |  |  |  |  |  |  |
|  | 4.3 Monitoring instruments | 4.3.1 Master the correct operating procedures for multi-parameter monitors ，including analysis and management of various alarms |  |  |  |  |  |  |
|  |  | 4.3.2 Familiar with the standard operating procedures for cardiac output monitors |  |  |  |  |  |  |
|  |  | 4.3.3 Master the standard operating procedures of BIS monitors |  |  |  |  |  |  |
|  |  | 4.3.4 Familiar with the standard operating procedures of cerebral oximetry monitors |  |  |  |  |  |  |
|  |  | 4.3.5 Know the correct operating procedures for neuromuscular monitoring devices |  |  |  |  |  |  |
|  | 4.4 Endotracheal intubation auxiliary equipment | 4.4.1 Master the structure of conventional laryngoscopes and the correct methods for assembling and disassembling blades. |  |  |  |  |  |  |
|  |  | 4.4.2 Master the structure of common video laryngoscopes and the correct methods for installing and removing blades |  |  |  |  |  |  |
|  |  | 4.4.3 Master the structure of fiberoptic bronchoscopes |  |  |  |  |  |  |
|  | 4.5 Medical infusion devices | 4.5.1 Master the correct operation and troubleshooting methods of syringe pumps and infusion pumps |  |  |  |  |  |  |
|  |  | 4.5.2 Master the correct operating methods and troubleshooting techniques for analgesia pumps |  |  |  |  |  |  |
|  | 4.6 Perioperative warming devices | 4.6.1 Master the correct operation and troubleshooting of conductive heating devices (e.g., circulating water blankets, electric heating blankets) |  |  |  |  |  |  |
|  |  | 4.6.2 Master the correct operating procedures and troubleshooting techniques for forced-air warming devices |  |  |  |  |  |  |
|  |  | 4.6.3 Master the correct operating procedures and troubleshooting methods for fluid warming devices |  |  |  |  |  |  |
|  | 4.7 Other special equipment | 4.7.1 Know the structure and application of blood salvage equipment |  |  |  |  |  |  |
|  |  | 4.7.2 Know the structure and operation of extracorporeal circulation equipment |  |  |  |  |  |  |
| Supplementary Indicators |  |  |  |  |  |  |  |  |
|  |  |  |  |  |  |  |  |  |
| 5. Infection prevention and control | 13. 14. 15. 16. 17. 18.   5.1 Personal protection | 5.1.1 Be able to select appropriate cleaning and disinfection devices and perform correct hand hygiene practices |  |  |  |  |  |  |
|  |  | 5.1.2 Be able to implement standard precautions correctly according to specific clinical situations |  |  |  |  |  |  |
|  | 5.2 Disinfection and isolation | 5.2.1 Know the disinfection management of commonly used instruments and supplies in the anesthesiology department |  |  |  |  |  |  |
|  |  | 5.2.2 Be able to properly dispose of medical waste, including sharps |  |  |  |  |  |  |
|  | 5.3 Aseptic technique | 5.3.1 Master aseptic principles |  |  |  |  |  |  |
|  |  | 5.3.2 Master commonly used aseptic practice methods |  |  |  |  |  |  |
| Supplementary Indicators |  |  |  |  |  |  |  |  |
|  |  |  |  |  |  |  |  |  |
| 6. Anesthesia documentation | 6.1 Anesthesia Records and PACU records | 6.1.1 Be able to complete anesthesia records in a timely, comprehensive and accurate manner. |  |  |  |  |  |  |
|  |  | 6.1.2 Be able to complete PACU records in a timely, comprehensive and accurate manner |  |  |  |  |  |  |
|  | 6.2 Other documentation | 6.2.1 Be able to fill out postoperative handover forms completely and accurately |  |  |  |  |  |  |
|  |  | 6.2.2 Be able to document postoperative analgesia follow‑up records in a timely, complete and accurate manner |  |  |  |  |  |  |
| Supplementary Indicators |  |  |  |  |  |  |  |  |
|  |  |  |  |  |  |  |  |  |
| 7. Risk management | 19. 20. 21. 22. 23. 24.   7.1 Surgical safety check | 7.1.1 Familiar with the timing of surgical safety checks |  |  |  |  |  |  |
|  |  | 7.1.2 Know the content of surgical safety checks |  |  |  |  |  |  |
|  | 7.2 Anesthesia quality management | 7.2.1 Familiar with the types and definitions of anesthesia quality indicators |  |  |  |  |  |  |
|  |  | 7.2.2 Familiar with the thresholds of various anesthesia quality indicators |  |  |  |  |  |  |
|  | 7.3 Anesthesia nursing quality management | 7.3.1 Know rules and regulations related to anesthesia nursing |  |  |  |  |  |  |
|  |  | 7.3.2 Familiar with standard operating procedures for anesthesia nursing |  |  |  |  |  |  |
|  |  | 7.3.3 Master the categories and definitions of quality control indicators for anesthesia nursing |  |  |  |  |  |  |
|  |  | 7.3.4 Master common quality management methods |  |  |  |  |  |  |
|  | 7.4 Adverse nursing events | 7.4.1 Master the classification of adverse nursing events |  |  |  |  |  |  |
|  |  | 7.4.2 Master risk assessment for various adverse nursing events |  |  |  |  |  |  |
|  |  | 7.4.3 Master preventive measures for all types of adverse nursing events |  |  |  |  |  |  |
|  |  | 7.4.4 Master the handling and reporting procedures of adverse nursing events |  |  |  |  |  |  |
|  | 7.5 Management of high-risk medications in anesthesiology | 7.5.1 Master the classification and management requirements of narcotic and psychotropic drugs, including storage, requisition, administration and return procedures |  |  |  |  |  |  |
|  |  | 7.5.2 Master the classification and management requirements of other high-risk medications in the anesthesiology department, including storage, requisition, administration, and return procedures |  |  |  |  |  |  |
| Supplementary Indicators |  |  |  |  |  |  |  |  |
|  |  |  |  |  |  |  |  |  |
| 8. Clinical nursing Research | 8.1 Evidence-based clinical translation | 8.1.1 Be able to formulate appropriate search strategies for evidence retrieval |  |  |  |  |  |  |
|  |  | 8.1.2 Be able to conduct comprehensive literature evaluation and provide valuable evidence-based references |  |  |  |  |  |  |
|  |  | 8.1.3 Be able to apply valid evidence in anesthesia nursing practice |  |  |  |  |  |  |
|  |  | 8.1.4 Be able to put forward suggestions for formulating evidence‑based nursing practice protocols and quality standards after evaluating the application scenarios of evidence |  |  |  |  |  |  |
|  | 8.2 Original research | 8.2.1 Possess the ability to actively transform clinical problems into research questions |  |  |  |  |  |  |
|  |  | 8.2.2 Be able to select appropriate research methods and statistical approaches for research design, conduct clinical studies, generate research outcomes, and propose recommendations for further investigations |  |  |  |  |  |  |
| Supplementary Indicators |  |  |  |  |  |  |  |  |
|  |  |  |  |  |  |  |  |  |
