# Supplementary material for: Core clinical competency indicators for master of nursing specialist (anaesthesia) students in China: a delphi study
Source: BMC Nurs. 2026 Jul 15;25:947. doi: 10.1186/s12912-026-05018-8 (PMC13651016; doi:10.1186/s12912-026-05018-8)
Supplement: Supplementary file 5 [file 12912_2026_5018_MOESM5_ESM.docx]

### [Additional](https://static-content.springer.com/esm/art:10.1186/s12912-024-01778-3/MediaObjects/12912_2024_1778_MOESM1_ESM.docx) **[file 5](https://static-content.springer.com/esm/art:10.1186/s12912-024-01778-3/MediaObjects/12912_2024_1778_MOESM1_ESM.docx)**

Table1 The score table indicating expert familiarity

| Degree of familiarity | Most Familiar | More familiar | Familiar | Less familiar | Unfamiliar |
| --- | --- | --- | --- | --- | --- |
| Expert self-evaluation | 1.0 | 0.8 | 0.6 | 0.4 | 0.2 |

Table2 Expert judgment basis score table

| Basis for making judgments | The extent of the impact on the significance of the indicators | | |
| --- | --- | --- | --- |
|  | Significant | intermediate | Insignificance |
| Theoretical analysis | 0.3 | 0.2 | 0.1 |
| Practical experi ence in the field | 0.5 | 0.4 | 0.3 |
| reference documentation | 0.1 | 0.1 | 0.1 |
| Intuitive Judgment | 0.1 | 0.1 | 0.1 |
