# Supplementary material for: Core clinical competency indicators for master of nursing specialist (anaesthesia) students in China: a delphi study
Source: BMC Nurs. 2026 Jul 15;25:947. doi: 10.1186/s12912-026-05018-8 (PMC13651016; doi:10.1186/s12912-026-05018-8)
Supplement: Supplementary file 6 [file 12912_2026_5018_MOESM6_ESM.docx]

### [Additional](https://static-content.springer.com/esm/art:10.1186/s12912-024-01778-3/MediaObjects/12912_2024_1778_MOESM1_ESM.docx) **[file 6](https://static-content.springer.com/esm/art:10.1186/s12912-024-01778-3/MediaObjects/12912_2024_1778_MOESM1_ESM.docx)**

### the Core clinical competency indicators for Master of Nursing Specialist (anaesthesia) students

| Indicators | Mean ± SD | CV | Proportion scored ≥ 4(%) |
| --- | --- | --- | --- |
| 1. Pre-anesthesia nursing assessment | 4.9±0.31 | 0.06 | 100% |
| 1.1 General information assessment | 4.75±0.44 | 0.09 | 100% |
| 1.1.1 Know patients’ basic information, including gender, age, ethnicity, place of birth, education level and occupation | 4.45±0.69 | 0.15 | 90% |
| 1.1.2 Know patients’ privacy needs, family support and religious beliefs | 4.4±0.75 | 0.17 | 85% |
| 1.2 Physical status assessment | 4.8±0.41 | 0.09 | 100% |
| 1.2.1 Know the patient's height, weight and vital signs | 4.7±0.47 | 0.10 | 100% |
| 1.2.2 Know positive signs of physical examination | 4.7±0.66 | 0.14 | 90% |
| 1.3 Comprehensive medical history assessment | 4.85±0.37 | 0.08 | 100% |
| 1.3.1 Know the patient's current medical history | 4.85±0.37 | 0.08 | 100% |
| 1.3.2 Know the patient's past medical history | 4.8±0.41 | 0.09 | 100% |
| 1.3.3 Know the positive indicators in the results of imaging examinations and laboratory tests | 4.75±0.44 | 0.09 | 100% |
| 1.3.4 Know the assessment of preoperative therapeutic medications | 4.75±0.44 | 0.09 | 100% |
| 1.4 Risk assessment | 4.9±0.31 | 0.06 | 100% |
| 1.4.1 Familiar with ASA classification | 4.65±0.59 | 0.13 | 95% |
| 1.4.2 Possess the ability to assess patients using relevant scales, such as those for falls, pressure injuries, and frailty | 4.75±0.44 | 0.09 | 100% |
| 1.5 Pre-anesthesia nursing plan | 4.85±0.37 | 0.08 | 100% |
| 1.5.1 Possess the ability to formulate an anesthesia nursing plan based on Pre-anesthesia nursing assessment results, combined with current knowledge, concepts, available evidence, and nursing principles | 4.45±0.76 | 0.17 | 85% |
| 2. Clinical anesthesia nursing | 4.9±0.31 | 0.06 | 100% |
| 2.1 Pre-anesthesia preparation | 4.75±0.44 | 0.09 | 100% |
| 2.1.1 Master the preoperative medication preparation for various anesthesia methods | 4.5±0.69 | 0.15 | 90% |
| 2.1.2 Master the preparation of preoperative supplies for various anesthesia methods | 4.55±0.60 | 0.13 | 95% |
| 2.2 Knowledge of drugs during the Peri-anesthesia period | 4.55±0.51 | 0.11 | 100% |
| 2.2.1 Familiar with the pharmacological effects, dosage, administration methods and adverse reactions of various anesthetic drugs | 4.55±0.76 | 0.17 | 85% |
| 2.2.2 Familiar with the pharmacological effects, dosage, administration methods and adverse reactions of various analgesic drugs | 4.55±0.76 | 0.17 | 85% |
| 2.2.3 Familiar with the pharmacological effects, dosage, administration methods and adverse reactions of various muscle relaxants | 4.55±0.76 | 0.17 | 85% |
| 2.2.4 Familiar with the pharmacological effects, dosage, administration methods and adverse reactions of various first-aid drugs | 4.65±0.67 | 0.14 | 90% |
| 2.2.5 Familiar with the pharmacological effects, dosage, administration methods and adverse reactions of other perioperative adjuvant drugs | 4.5±0.76 | 0.17 | 85% |
| 2.3 Airway management | 4.75±0.55 | 0.12 | 95% |
| 2.3.1 Master mask ventilation techniques, including EC clamp technique and two-hand mask ventilation technique | 4.8±0.41 | 0.09 | 100% |
| 2.3.2 Master the insertion techniques of oropharyngeal airways and nasopharyngeal airways | 4.75±0.55 | 0.12 | 95% |
| 2.3.3 Familiar with the structure of supraglottic airway devices and their insertion operation procedures, and be able to assist in completing the insertion | 4.6±0.68 | 0.15 | 90% |
| 2.3.4 Familiar with the structure and insertion procedures of various endotracheal tubes and bronchial tubes, and be able to assist in completing the insertion | 4.55±0.69 | 0.15 | 90% |
| 2.3.5 Familiar with the difficult airway management process | 4.55±0.76 | 0.17 | 85% |
| 2.4 Assistance with various anesthesia methods | 4.7±0.57 | 0.12 | 95% |
| 2.4.1 Master the principles, complications, and processes of general anesthesia, and be able to assist anesthesiologists in performing general anesthesia | 4.6±0.68 | 0.15 | 90% |
| 2.4.2 Master the principles and complications of neuraxial anesthesia, and be able to assist anesthesiologists in performing neuraxial anesthesia | 4.55±0.69 | 0.15 | 90% |
| 2.4.3 Master the principles and complications of peripheral nerve block, and be able to assist anesthesiologists in performing peripheral nerve block | 4.5±0.76 | 0.17 | 85% |
| 2.5 Anesthesia nursing for various specialist surgeries | 4.8±0.52 | 0.11 | 95% |
| 2.5.1 Familiar with the characteristics of surgeries in various specialties and their impacts on anesthesia | 4.35±0.75 | 0.17 | 85% |
| 2.5.2 Familiar with anesthetic management of common surgeries across various specialties | 4.5±0.76 | 0.17 | 85% |
| 2.5.3 Be able to assist in correctly positioning surgical patients, maintain unobstructed pipelines, and protect compressed skin areas and nerve plexuses. | 4.75±0.44 | 0.09 | 100% |
| 2.5.4 Possess the ability to comprehensively evaluate the patient's condition based on various monitoring indicators, identify potential risks endangering patient safety, and implement appropriate countermeasures | 4.65±0.59 | 0.13 | 95% |
| 2.5.5 Be able to conduct dynamic assessment and adjustment based on various data while implementing the anesthesia nursing plan | 4.75±0.55 | 0.12 | 95% |
| 2.5.6 Be able to employ appropriate communication skills, provide psychological support, and help patients get through the perioperative period | 4.8±0.52 | 0.11 | 95% |
| 2.6 Anesthetic Crisis Recognition and Management | 4.75±0.44 | 0.09 | 100% |
| 2.6.1 Be able to rapidly assess emergent critical incidents, deliver urgent interventions within the scope of professional responsibilities, and activate emergency protocols when necessary | 4.7±0.57 | 0.12 | 95% |
| 2.6.2 Be able to promptly seek assistance from operating room nurses, anesthesiologists, surgeons, and other staff, and actively cooperate with the team in emergency treatment | 4.9±0.31 | 0.06 | 100% |
| 2.7 Safe transfer and handover of postoperative patients | 4.95±0.22 | 0.05 | 100% |
| 2.7.1 Master the pre-transfer assessment of postoperative patients | 4.8±0.41 | 0.09 | 100% |
| 2.7.2 Master all preparations (including medications, supplies, instruments and equipment) prior to the transfer of postoperative patients | 4.75±0.44 | 0.09 | 100% |
| 2.7.3 Familiar with the procedures and key focuses for the transfer of postoperative patients | 4.85±0.37 | 0.08 | 100% |
| 2.7.4 Familiar with emergency management of unexpected incidents during the transfer of postoperative patients | 4.9±0.31 | 0.06 | 100% |
| 2.7.5 Master the content and procedures of handover with nurses from the PACU, ICU, and general wards | 4.85±0.37 | 0.08 | 100% |
| 2.8 Monitoring and nursing in the PACU | 4.95±0.22 | 0.05 | 100% |
| 2.8.1 Master the management regulations and routine work protocols of the PACU | 4.9±0.31 | 0.06 | 100% |
| 2.8.2 Master the post-anesthesia nursing characteristics of various specialized surgeries, as well as the assessment and nursing care of common anesthetic complications | 4.85±0.37 | 0.08 | 100% |
| 2.8.3 Master the early postoperative nursing processes, key points, and the assessment and nursing of complications for various specialty surgeries | 4.85±0.37 | 0.08 | 100% |
| 2.8.4 Master risk identification and management during the anesthesia recovery period | 4.85±0.37 | 0.08 | 100% |
| 2.9 Pain Management | 4.75±0.44 | 0.09 | 100% |
| 2.9.1 Know the principles of perioperative acute pain management | 4.7±0.47 | 0.10 | 100% |
| 2.9.2 Master commonly used pain assessment methods | 4.8±0.52 | 0.11 | 95% |
| 2.9.3 Master nursing care for surgical patients' pain, assist in implementing pain interventions, and promptly manage complications related to analgesic therapy | 4.75±0.55 | 0.12 | 95% |
| 2.9.4 Master the follow-up content and workflow of postoperative analgesia | 4.8±0.41 | 0.09 | 100% |
| 3. Anesthesia Monitoring | 4.8±0.52 | 0.11 | 95% |
| 3.1 Circulatory system monitoring | 4.75±0.55 | 0.12 | 95% |
| 3.1.1 Master the monitoring methods of electrocardiography (ECG), grasp the characteristics of normal ECG, and be familiar with the ECG manifestations of common arrhythmias | 4.65±0.67 | 0.14 | 90% |
| 3.1.2 Master the monitoring methods and normal reference ranges for common arterial and venous pressure parameters (including NBP, ABP, CVP, etc.) | 4.65±0.67 | 0.14 | 90% |
| 3.1.3 Master the principles and methods of pulmonary artery pressure monitoring | 4.35±0.81 | 0.19 | 80% |
| 3.1.4 Master invasive monitoring techniques for cardiac output | 4.5±0.76 | 0.17 | 85% |
| 3.2 Respiratory function monitoring | 4.65±0.67 | 0.14 | 90% |
| 3.2.1 Familiar with the monitoring and normal reference ranges of respiratory parameters (tidal volume, respiratory rate, airway pressure, end-tidal carbon dioxide, etc.) | 4.8±0.52 | 0.11 | 95% |
| 3.2.2 Master the monitoring method and normal reference range of pulse oximetry saturation | 4.75±0.55 | 0.12 | 95% |
| 3.3 Neurological monitoring | 4.55±0.76 | 0.17 | 85% |
| 3.3.1 Master the monitoring methods and normal reference ranges of Bispectral Index (BIS) | 4.6±0.75 | 0.16 | 85% |
| 3.3.2 Familiar with the monitoring methods and normal reference ranges of regional cerebral oxygen saturation (rSO₂) | 4.5±0.76 | 0.17 | 85% |
| 3.4 Temperature monitoring | 4.75±0.55 | 0.12 | 95% |
| 3.4.1 Master the methods and normal reference ranges of perioperative temperature monitoring | 4.75±0.44 | 0.09 | 100% |
| 3.4.2 Master the methods of maintaining normal body temperature in perioperative care | 4.8±0.41 | 0.09 | 100% |
| 3.5 Intake and output monitoring | 4.75±0.55 | 0.12 | 95% |
| 3.5.1 Master the content and methods of intake monitoring | 4.85±0.37 | 0.08 | 100% |
| 3.5.2 Master the content and methods of output monitoring | 4.85±0.37 | 0.08 | 100% |
| 3.6 Neuromuscular transmission function monitoring | 4.25±1.02 | 0.24 | 80% |
| 3.6.1 Know monitoring methods of neuromuscular function | 4.05±1.00 | 0.25 | 75% |
| 4. Application of instruments and equipment | 4.65±0.67 | 0.14 | 90% |
| 4.1 Anesthesia machine | 4.7±0.66 | 0.14 | 90% |
| 4.1.1 Master the self-check and routine operation of anesthesia machines, including analysis and management of common alarms | 4.7±0.66 | 0.14 | 90% |
| 4.1.2 Know the causes and management of common faults of anesthesia machines | 4.6±0.75 | 0.16 | 85% |
| 4.2 Ventilator | 4.7±0.66 | 0.14 | 90% |
| 4.2.1 Master the self-check and routine operation of ventilators, including the analysis and management of common alarms | 4.6±0.82 | 0.18 | 90% |
| 4.2.2 Know the causes and management of common ventilator malfunctions | 4.65±0.81 | 0.17 | 90% |
| 4.3 Monitoring instruments | 4.75±0.55 | 0.12 | 95% |
| 4.3.1 Master the correct operating procedures for multi-parameter monitors ，including analysis and management of various alarms | 4.7±0.66 | 0.14 | 90% |
| 4.3.2 Familiar with the standard operating procedures for cardiac output monitors | 4.45±0.83 | 0.19 | 90% |
| 4.3.3 Master the standard operating procedures of BIS monitors | 4.5±0.83 | 0.18 | 90% |
| 4.3.4 Familiar with the standard operating procedures of cerebral oximetry monitors | 4.4±0.82 | 0.19 | 90% |
| 4.3.5 Know the correct operating procedures for neuromuscular monitoring devices | 4.45±0.83 | 0.19 | 90% |
| 4.4 Endotracheal intubation auxiliary equipment | 4.65±0.59 | 0.13 | 95% |
| 4.4.1 Master the structure of conventional laryngoscopes and the correct methods for assembling and disassembling blades. | 4.7±0.57 | 0.12 | 95% |
| 4.4.2 Master the structure of common video laryngoscopes and the correct methods for installing and removing blades | 4.65±0.67 | 0.14 | 90% |
| 4.4.3 Master the structure of fiberoptic bronchoscopes | 4.55±0.83 | 0.18 | 90% |
| 4.5 Medical infusion devices | 4.7±0.57 | 0.12 | 95% |
| 4.5.1 Master the correct operation and troubleshooting methods of syringe pumps and infusion pumps | 4.75±0.44 | 0.09 | 100% |
| 4.5.2 Master the correct operating methods and troubleshooting techniques for analgesia pumps | 4.8±0.41 | 0.09 | 100% |
| 4.6 Perioperative warming devices | 4.7±0.57 | 0.12 | 95% |
| 4.6.1 Master the correct operation and troubleshooting of conductive heating devices (e.g., circulating water blankets, electric heating blankets) | 4.5±0.83 | 0.18 | 90% |
| 4.6.2 Master the correct operating procedures and troubleshooting techniques for forced-air warming devices | 4.65±0.59 | 0.13 | 95% |
| 4.6.3 Master the correct operating procedures and troubleshooting methods for fluid warming devices | 4.65±0.59 | 0.13 | 95% |
| 4.7 Other special equipment | 4.25±0.72 | 0.17 | 85% |
| 4.7.1 Know the structure and application of blood salvage equipment | 4.5±0.61 | 0.13 | 95% |
| 4.7.2 Know the structure and operation of extracorporeal circulation equipment | 4.25±0.72 | 0.17 | 85% |
| 5. Infection prevention and control | 4.95±0.22 | 0.05 | 100% |
| 5.1 Personal protection | 4.9±0.31 | 0.06 | 100% |
| 5.1.1 Be able to select appropriate cleaning and disinfection devices and perform correct hand hygiene practices | 4.9±0.31 | 0.06 | 100% |
| 5.1.2 Be able to implement standard precautions correctly according to specific clinical situations | 4.9±0.31 | 0.06 | 100% |
| 5.2 Disinfection and isolation | 4.9±0.31 | 0.06 | 100% |
| 5.2.1 Know the disinfection management of commonly used instruments and supplies in the anesthesiology department | 4.85±0.37 | 0.08 | 100% |
| 5.2.2 Be able to properly dispose of medical waste, including sharps | 4.8±0.41 | 0.09 | 100% |
| 5.3 Aseptic technique | 4.85±0.37 | 0.08 | 100% |
| 5.3.1 Master aseptic principles | 4.95±0.22 | 0.05 | 100% |
| 5.3.2 Master commonly used aseptic practice methods | 4.95±0.22 | 0.05 | 100% |
| 6. Anesthesia documentation | 4.05±0.76 | 0.19 | 75% |
| 6.1 Anesthesia Records and PACU records | 4.4±0.94 | 0.21 | 95% |
| 6.1.1 Be able to complete anesthesia records in a timely, comprehensive and accurate manner. | 4.6±0.68 | 0.15 | 90% |
| 6.1.2 Be able to complete PACU records in a timely, comprehensive and accurate manner | 4.7±0.57 | 0.12 | 95% |
| 6.2 Other documentation | 4.05±0.83 | 0.20 | 80% |
| 6.2.1 Be able to fill out postoperative handover forms completely and accurately | 4.75±0.55 | 0.12 | 95% |
| 6.2.2 Be able to document postoperative analgesia follow‑up records in a timely, complete and accurate manner | 4.6±0.60 | 0.13 | 95% |
| 7. Risk management | 4.8±0.52 | 0.11 | 95% |
| 7.1 Surgical safety check | 4.95±0.22 | 0.05 | 100% |
| 7.1.1 Familiar with the timing of surgical safety checks | 4.8±0.52 | 0.11 | 95% |
| 7.1.2 Know the content of surgical safety checks | 4.85±0.49 | 0.10 | 95% |
| 7.2 Anesthesia quality management | 4.5±0.83 | 0.18 | 90% |
| 7.2.1 Familiar with the types and definitions of anesthesia quality indicators | 4.65±0.59 | 0.13 | 95% |
| 7.2.2 Familiar with the thresholds of various anesthesia quality indicators | 4.45±0.69 | 0.15 | 90% |
| 7.3 Anesthesia nursing quality management | 4.85±0.49 | 0.10 | 95% |
| 7.3.1 Know rules and regulations related to anesthesia nursing | 4.8±0.41 | 0.09 | 100% |
| 7.3.2 Familiar with standard operating procedures for anesthesia nursing | 4.85±0.37 | 0.08 | 100% |
| 7.3.3 Master the categories and definitions of quality control indicators for anesthesia nursing | 4.7±0.57 | 0.12 | 95% |
| 7.3.4 Master common quality management methods | 4.45±0.76 | 0.17 | 85% |
| 7.4 Adverse nursing events | 4.9±0.45 | 0.09 | 95% |
| 7.4.1 Master the classification of adverse nursing events | 4.75±0.44 | 0.09 | 100% |
| 7.4.2 Master risk assessment for various adverse nursing events | 4.75±0.55 | 0.12 | 95% |
| 7.4.3 Master preventive measures for all types of adverse nursing events | 4.8±0.41 | 0.09 | 100% |
| 7.4.4 Master the handling and reporting procedures of adverse nursing events | 4.85±0.37 | 0.08 | 100% |
| 7.5 Management of high-risk medications in anesthesiology | 4.9±0.45 | 0.09 | 95% |
| 7.5.1 Master the classification and management requirements of narcotic and psychotropic drugs, including storage, requisition, administration and return procedures | 4.85±0.37 | 0.08 | 100% |
| 7.5.2 Master the classification and management requirements of other high-risk medications in the anesthesiology department, including storage, requisition, administration, and return procedures | 4.8±0.41 | 0.09 | 100% |
| 8. Clinical nursing Research | 4.2±0.70 | 0.17 | 95% |
| 8.1 Evidence-based clinical translation | 4.5±0.69 | 0.15 | 90% |
| 8.1.1 Be able to formulate appropriate search strategies for evidence retrieval | 4.65±0.67 | 0.14 | 90% |
| 8.1.2 Be able to conduct comprehensive literature evaluation and provide valuable evidence-based references | 4.55±0.69 | 0.15 | 90% |
| 8.1.3 Be able to apply valid evidence in anesthesia nursing practice | 4.65±0.67 | 0.14 | 90% |
| 8.1.4 Be able to put forward suggestions for formulating evidence‑based nursing practice protocols and quality standards after evaluating the application scenarios of evidence | 4.5±0.69 | 0.15 | 90% |
| 8.2 Original research | 4.55±0.60 | 0.13 | 95% |
| 8.2.1 Possess the ability to actively transform clinical problems into research questions | 4.65±0.67 | 0.14 | 90% |
| 8.2.2 Be able to select appropriate research methods and statistical approaches for research design, conduct clinical studies, generate research outcomes, and propose recommendations for further investigations | 4.55±0.69 | 0.15 | 90% |

**Note. Indicator 3.6.1 had borderline CV (0.25) and mean (4.05); retained because experts commented that neuromuscular monitoring is emerging in Chinese anesthesia nursing.**
