# Supplementary material for: Core clinical competency indicators for master of nursing specialist (anaesthesia) students in China: a delphi study
Source: BMC Nurs. 2026 Jul 15;25:947. doi: 10.1186/s12912-026-05018-8 (PMC13651016; doi:10.1186/s12912-026-05018-8)
Supplement: Supplementary file 7 [file 12912_2026_5018_MOESM7_ESM.docx]

### [Additional](https://static-content.springer.com/esm/art:10.1186/s12912-024-01778-3/MediaObjects/12912_2024_1778_MOESM1_ESM.docx) **[file 7](https://static-content.springer.com/esm/art:10.1186/s12912-024-01778-3/MediaObjects/12912_2024_1778_MOESM1_ESM.docx)**

### Comparison of original indicators and revised indicators in the Round 1

**Table 1 The primary indicators**

| **Primary indicators** | **Revision type** | **Revised indicators** |
| --- | --- | --- |
| 1.Pre-anesthesia health assessment | rename | Pre-anesthesia nursing assessment |
| 2.Clinical anesthesia nursing management | merge & rename | Clinical anesthesia nursing |
| 5.Termination of anesthesia |  |  |
| 6.Postoperative nursing and pain management |  |  |
| 3. Anesthesia Monitoring |  |  |
| 4. Equipment | rename | Application of equipment |
| 7.Infection and control | rename | Infection prevention and control |
| 8.Anesthesia specialty documentation | rename | Anesthesia documentation |
| 9.Risk management |  |  |
| 10.Clinical nursing Research |  |  |

**Table 2	The secondary indicators**

| **Secondary indicators** | **Revision type** | **Revised indicators** |
| --- | --- | --- |
| 1.1 Comprehensive medical history assessment |  |  |
| 1.2 Pre-anesthesia nursing plan |  |  |
| 2.1 Pre-anesthesia preparation |  |  |
| 2.2 Peri-anesthetic Medications | rename | Knowledge of Peri-anesthetic Medications |
| 2.3 Airway management |  |  |
| 2.4 Various Anesthesia Methods | rename | Assistance with Various Anesthesia Methods |
| 2.5 Anesthesia nursing for various specialist surgeries |  |  |
| 2.6 Anesthetic Crisis Recognition and Management |  |  |
| 1. 2. 3.   3.1 ECG and Hemodynamic Monitoring | rename | Circulatory System Monitoring |
| 3.2 Respiratory Gas Exchange Monitoring | rename | Respiratory Function Monitoring |
| 3.3 Neurological monitoring |  |  |
| 3.4 Other Monitoring | delete |  |
| 1. 2. 3. 4.   4.1 Anesthesia machine |  |  |
| 4.2 Ventilator |  |  |
| 4.3 Monitoring instruments |  |  |
| 4.4 Endotracheal intubation auxiliary equipment |  |  |
| 4.5 Other special equipment |  |  |
| 5.1 Safe transfer of post-anesthesia patients | merge | Safe transfer and handover of Post-anesthesia patients |
| 5.2 Handover of post-anesthesia patients |  |  |
| 1. 2. 3. 4. 5. 6.   6.1 Post-Anesthesia Recovery Nursing | rename | Monitoring and Nursing in the Post-Anesthesia Care Unit (PACU) |
| 6.2 Pain Management |  |  |
| 7. 8. 9. 10. 11. 12.   7.1 Personal protection |  |  |
| 7.2 Disinfection and isolation |  |  |
| 7.3 Aseptic technique |  |  |
| 8.1 Anesthesia Records and PACU records |  |  |
| 8.2 Other documentation |  |  |
| 13. 14. 15. 16. 17. 18.   9.1 Surgical safety check |  |  |
| 9.2 Anesthesia quality management |  |  |
| 9.3 Anesthesia nursing quality management |  |  |
| 9.4 Adverse nursing events |  |  |
| 9.5 Management of Narcotic Drugs | rename | Management of High-Risk Medications in Anesthesiology |
| 10.1 Evidence-based clinical translation |  |  |
| 10.2 Clinical Research Ability | rename | Original Research |
|  | add | General information assessment |
|  | add | Physical status assessment |
|  | add | Risk Assessment |
|  | add | Body temperature monitoring |
|  | add | Intake and output monitoring |
|  | add | Neuromuscular transmission function monitoring |
|  | add | Medical infusion devices |
|  | add | Perioperative warming devices |

**Table 3 The tertiary indicators**

| **Tertiary indicators** | **Revision type** | **Revised indicators** |
| --- | --- | --- |
| 1.1.1 Know the patient's height, weight and vital signs |  |  |
| 1.1.2 Know positive signs of physical examination |  |  |
| 1.1.3 Know the patient's current medical history |  |  |
| 1.1.4 Know the patient's past medical history |  |  |
| 1.1.5 Know the positive indicators in the results of imaging examinations and laboratory tests |  |  |
| 1.1.6 Know the assessment of preoperative therapeutic medications |  |  |
| 1.1.7 Familiar with ASA classification |  |  |
| 1.2.1 Possess the ability to formulate an anesthesia nursing plan based on Pre-anesthesia nursing assessment results, combined with current knowledge, concepts, available evidence, and nursing principles |  |  |
| 1.2.2 Assessing patients’ psychological status and providing timely psychological care | delete |  |
| 2.1.1 Master the preoperative medication preparation for various anesthesia methods |  |  |
| 2.1.2 Master the preparation of preoperative supplies for various anesthesia methods |  |  |
| 2.2.1 Familiar with the pharmacological effects, dosage, administration methods and adverse reactions of various anesthetic drugs |  |  |
| 2.2.2 Familiar with the pharmacological effects, dosage, administration methods and adverse reactions of various analgesic drugs |  |  |
| 2.2.3 Familiar with the pharmacological effects, dosage, administration methods and adverse reactions of various muscle relaxants |  |  |
| 2.2.4 Familiar with the pharmacological effects, dosage, administration methods and adverse reactions of various first-aid drugs |  |  |
| 2.2.5 Familiar with the pharmacological effects, dosage, administration methods and adverse reactions of other perioperative adjuvant drugs |  |  |
| 2.3.1 Master mask ventilation techniques, including EC clamp technique and two-hand mask ventilation technique |  |  |
| 2.3.2 Master the insertion techniques of oropharyngeal airways and nasopharyngeal airways |  |  |
| 2.3.3 Familiar with the structure of supraglottic airway devices and their insertion operation procedures, and be able to assist in completing the insertion |  |  |
| 2.3.4 Familiar with the structure and insertion procedures of various endotracheal tubes and bronchial tubes, and be able to assist in completing the insertion |  |  |
| 2.3.5 Familiar with the difficult airway management process |  |  |
| 2.4.1 Master the principles, complications, and processes of general anesthesia, and be able to assist anesthesiologists in performing general anesthesia |  |  |
| 2.4.2 Master the principles and complications of neuraxial anesthesia, and be able to assist anesthesiologists in performing neuraxial anesthesia |  |  |
| 2.4.3 Master the principles and complications of peripheral nerve block, and be able to assist anesthesiologists in performing peripheral nerve block |  |  |
| 2.5.1 Familiar with the characteristics of surgeries in various specialties and their impacts on anesthesia |  |  |
| 2.5.2 Familiar with anesthetic management of common surgeries across various specialties |  |  |
| 2.5.3 Be able to assist in correctly positioning surgical patients, maintain unobstructed pipelines, and protect compressed skin areas and nerve plexuses. |  |  |
| 2.5.4 Possess the ability to comprehensively evaluate the patient's condition based on various monitoring indicators, identify potential risks endangering patient safety, and implement appropriate countermeasures |  |  |
| 2.5.5 Be able to conduct dynamic assessment and adjustment based on various data while implementing the anesthesia nursing plan |  |  |
| 2.5.6 Be able to employ appropriate communication skills, provide psychological support, and help patients get through the perioperative period |  |  |
| 2.6.1 Be able to rapidly assess emergent critical incidents, deliver urgent interventions within the scope of professional responsibilities, and activate emergency protocols when necessary |  |  |
| 2.6.2 Be able to promptly seek assistance from operating room nurses, anesthesiologists, surgeons, and other staff, and actively cooperate with the team in emergency treatment |  |  |
| 3.1.1 Master the monitoring methods of electrocardiography (ECG), grasp the characteristics of normal ECG, and be familiar with the ECG manifestations of common arrhythmias |  |  |
| 3.1.2 Master the monitoring methods and normal reference ranges for common arterial and venous pressure parameters (including NBP, ABP, CVP, etc.) |  |  |
| 3.1.3 Master the principles and methods of pulmonary artery pressure monitoring |  |  |
| 3.1.4 Master invasive monitoring techniques for cardiac output |  |  |
| 3.2.1 Familiar with the monitoring and normal reference ranges of respiratory parameters (tidal volume, respiratory rate, airway pressure, end-tidal carbon dioxide, etc.) |  |  |
| 3.2.2 Master the monitoring method and normal reference range of pulse oximetry saturation |  |  |
| 3.3.1 Master the monitoring methods and normal reference ranges of Bispectral Index (BIS) |  |  |
| 3.3.2 Familiar with the monitoring methods and normal reference ranges of regional cerebral oxygen saturation (rSO₂) |  |  |
| 3.4.1 Master the methods and normal reference ranges of perioperative temperature monitoring |  |  |
| 3.4.2 Master the methods of maintaining normal body temperature in perioperative care |  |  |
| 3.4.3 Master urine output monitoring | rename | Master the content and methods of output monitoring |
| 3.4.4 Know monitoring methods of neuromuscular function |  |  |
| 4.1.1 Master the self-check and routine operation of anesthesia machines, including analysis and management of common alarms |  |  |
| 4.1.2 Know the causes and management of common faults of anesthesia machines |  |  |
| 4.2.1 Master the self-check and routine operation of ventilators, including the analysis and management of common alarms |  |  |
| 4.2.2 Know the causes and management of common ventilator malfunctions |  |  |
| 4.3.1 Master the correct operating procedures for multi-parameter monitors ，including analysis and management of various alarms |  |  |
| 4.3.2 Familiar with the standard operating procedures for cardiac output monitors |  |  |
| 4.3.3 Master the standard operating procedures of BIS monitors |  |  |
| 4.3.4 Familiar with the standard operating procedures of cerebral oximetry monitors |  |  |
| 4.3.5 Know the correct operating procedures for neuromuscular monitoring devices |  |  |
| 4.4.1 Master the structure of conventional laryngoscopes and the correct methods for assembling and disassembling blades. |  |  |
| 4.4.2 Master the structure of common video laryngoscopes and the correct methods for installing and removing blades |  |  |
| 4.4.3 Master the structure of fiberoptic bronchoscopes |  |  |
| 4.5.1 Master the correct operation and troubleshooting methods of syringe pumps and infusion pumps |  |  |
| 4.5.2 Master the correct operating methods and troubleshooting techniques for analgesia pumps |  |  |
| 4.5.3 Master the correct operation and troubleshooting of conductive heating devices (e.g., circulating water blankets, electric heating blankets) |  |  |
| 4.5.4 Master the correct operating procedures and troubleshooting techniques for forced-air warming devices |  |  |
| 4.5.5 Master the correct operating procedures and troubleshooting methods for fluid warming devices |  |  |
| 4.5.6 Know the structure and application of blood salvage equipment |  |  |
| 4.5.7 Know the structure and operation of extracorporeal circulation equipment |  |  |
| 5.1.1 Master the pre-transfer assessment of postoperative patients |  |  |
| 5.1.2 Master all preparations (including medications, supplies, instruments and equipment) prior to the transfer of postoperative patients |  |  |
| 5.1.3 Familiar with the procedures and key focuses for the transfer of postoperative patients |  |  |
| 5.2.1 Master the handover content with nurses from the PACU, ICU, and general wards | rename | Master the content and procedures of handover with nurses from the PACU, ICU, and general wards |
| 6.1.1 Master the management regulations and routine work protocols of the PACU |  |  |
| 6.1.2 Master the post-anesthesia nursing characteristics of various specialized surgeries, as well as the assessment and nursing care of common anesthetic complications |  |  |
| 6.1.3Master the postoperative nursing processes, as well as the assessment and nursing of complications for various specialty surgeries | rename | Master the early postoperative nursing processes, key points, and the assessment and nursing of complications for various specialty surgeries |
| 6.2.1 Know the principles of perioperative pain management |  |  |
| 6.2.2 Master commonly used pain assessment methods |  |  |
| 6.2.3 Master nursing care for surgical patients' pain, assist in implementing pain interventions, and promptly manage complications related to analgesic therapy |  |  |
| 6.2.4 Master the follow-up content and workflow of postoperative analgesia |  |  |
| 7.1.1 Be able to select appropriate cleaning and disinfection devices and perform correct hand hygiene practices |  |  |
| 7.1.2 Be able to implement standard precautions correctly according to specific clinical situations |  |  |
| 7.2.1 Know the disinfection management of commonly used instruments and supplies in the anesthesiology department |  |  |
| 7.2.2 Be able to properly dispose of medical waste, including sharps |  |  |
| 7.3.1 Master aseptic principles |  |  |
| 7.3.2 Master commonly used aseptic practice methods |  |  |
| 8.1.1 Be able to complete anesthesia records in a timely, comprehensive and accurate manner. |  |  |
| 8.1.2 Be able to complete PACU records in a timely, comprehensive and accurate manner |  |  |
| 8.2.1 Be able to fill out postoperative handover forms completely and accurately |  |  |
| 8.2.2 Be able to document postoperative analgesia follow‑up records in a timely, complete and accurate manner |  |  |
| 9.1.1 Familiar with the timing of surgical safety checks |  |  |
| 9.1.2 Know the content of surgical safety checks |  |  |
| 9.2.1 Know the types and definitions of anesthesia quality indicators | rename | Familiar with the types and definitions of anesthesia quality indicators |
| 9.2.2 Know the thresholds of various anesthesia quality indicators | rename | Familiar with the thresholds of various anesthesia quality indicators |
| 9.3.1 Know rules and regulations related to anesthesia nursing |  |  |
| 9.3.2 Familiar with standard operating procedures for anesthesia nursing |  |  |
| 9.3.3 Master the categories and definitions of quality control indicators for anesthesia nursing |  |  |
| 9.3.4 Know common quality management methods | rename | Master common quality management methods |
| 9.4.1 Master the classification of adverse nursing events |  |  |
| 9.4.2 Master risk assessment for various adverse nursing events |  |  |
| 9.4.3 Master preventive measures for all types of adverse nursing events |  |  |
| 9.4.4 Master the handling and reporting procedures of adverse nursing events |  |  |
| 9.5.1 Master the classification and management requirements of narcotic and psychotropic drugs, including storage, requisition, administration and return procedures |  |  |
| 9.5.2 Master the classification and management requirements of other high-risk medications in the anesthesiology department, including storage, requisition, administration, and return procedures |  |  |
| 10.1.1 Be able to formulate appropriate search strategies for evidence retrieval |  |  |
| 10.1.2 Be able to conduct comprehensive literature evaluation and provide valuable evidence-based references |  |  |
| 10.1.3 Be able to apply valid evidence in anesthesia nursing practice |  |  |
| 10.1.4 Be able to put forward suggestions for formulating evidence‑based nursing practice protocols and quality standards after evaluating the application scenarios of evidence |  |  |
| 10.2.1 Possess the ability to actively transform clinical problems into research questions |  |  |
| 10.2.2 Be able to select appropriate research methods and statistical approaches for research design, conduct clinical studies, generate research outcomes, and propose recommendations for further investigations |  |  |
|  | add | Know patients’ basic information, including gender, age, ethnicity, place of birth, education level and occupation |
|  | add | Know patients’ privacy needs, family support and religious beliefs |
|  | add | Ability to assess patients using relevant scales, such as those for falls, pressure injuries, and frailty |
|  | add | Familiarity with emergency handling procedures for unexpected events during the transfer of post-anesthesia patients |
|  | add | Risk identification and management during the anesthesia recovery period |
|  | add | Master the content and methods of intake monitoring |
